# Exploring the Therapeutic Potential of *Oenothera laciniata*: Antioxidant, Anti‐Hyperglycemic and Histopathological Activities Using In Vitro and In Vivo Approaches

**DOI:** 10.1002/fsn3.72095

**Published:** 2026-07-13

**Authors:** Dur‐E‐Najaf Khan, Syed Muhammad Mukarram Shah, Muska Mahabat Khan, Rehman Zafar, Muhammad Saeed Jan, Muhammad Ibrar, Abdur Rauf, Yahya S. Al‐Awthan, Omar S. Bahattab, Rakibur Rahman

**Affiliations:** ^1^ Department of Pharmacy University of Swabi Swabi KP Pakistan; ^2^ Department of Pharmacy Bacha Khan University Charsadda KP Pakistan; ^3^ Akson College of Pharmacy Mirpur University of Science and Technology Mirpur AJ & Kashmir Pakistan; ^4^ Department of Chemistry University of Swabi Swabi Khyber Pakhtunkhwa Pakistan; ^5^ Department of Biology, Faculty of Science University of Tabuk Tabuk Saudi Arabia; ^6^ Department of Pharmacy, Faculty of Science and Engineering International Islamic University Chittagong Chattogram Bangladesh

**Keywords:** antioxidant potential, GC–MS, in vitro, in vivo anti‐diabetic and histopathology, *Oenothera laciniata*

## Abstract

This study investigate the crude ethanolic extract of *Oenothera laciniata* (EOL) and its solvent fractions n‐hexane (NHOL), dichloromethane (DCMOL), ethyl acetate (EAOL), and aqueous (AQOL) for their phytochemical composition and antidiabetic potential through in vitro and in vivo analyses. GC–MS profiling revealed that the DCMOL fraction contained 8 compounds while the EAOL and NHOL fractions contained 19 and 20 compounds, respectively. Antioxidant evaluation using DPPH, ABTS, and H_2_O_2_ assays demonstrated that DCMOL exhibited the strongest free radical scavenging activity with the lowest IC_50_ values across all assays. Enzyme inhibition studies demonstrated that DCMOL, EAOL, and NHOL possessed significant α‐glucosidase and α‐amylase inhibitory activities, comparable to acarbose. In vivo studies confirmed that DCMOL significantly reduced blood glucose levels and improved glucose tolerance without inducing hepatotoxic or nephrotoxic effects. Histopathological analysis revealed partial restoration of pancreatic, hepatic, and renal architecture, particularly in the DCMOL and EAOL treated groups. *O. laciniata* , especially its DCMOL and EAOL fractions, exhibited strong antioxidant, enzyme inhibitory and antidiabetic activities, highlighting its potential as a natural multi‐target therapeutic candidate for diabetes management.

## Graphical abstract

Graphical abstract illustrating the therapeutic potential of *Oenothera laciniata* : antioxidant, anti‐hyperglycemic and histopathological activities using in vitro and in vivo approaches.

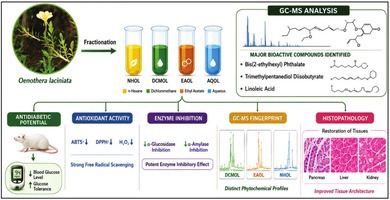

## 1 Introduction

Diabetes mellitus (DM) is a chronic metabolic disorder characterized by persistent hyperglycemia resulting from defects in insulin secretion, insulin action, or both (Jan et al. 2023). It is a global health challenge with an ever‐increasing prevalence, contributing significantly to morbidity and mortality worldwide (Khan, Rahman, et al. 2025). According to the International Diabetes Federation (IDF), the global prevalence of diabetes is projected to rise to 700 million by 2045, underscoring the urgent need for effective prevention and management strategies (Saeedi et al. 2019). DM is broadly classified into two types: Type 1 diabetes, caused by autoimmune destruction of pancreatic β‐cells, and Type 2 diabetes, which involves insulin resistance and relative insulin deficiency. The condition is associated with severe complications such as nephropathy, retinopathy, neuropathy, and cardiovascular diseases, leading to a reduced quality of life and increased healthcare burden (Fan et al. 2025). Currently, diabetes is treated with oral hypoglycemic agents and insulin. The oral hypoglycemic agents available in the pharmaceutical market include biguanides, pioglitazone, glibornuride, bromocriptine, glitazones, bezafibrate, glipizide, pioglitazone, rosiglitazone, saroglitazar, and metformin (Talib et al. 2023). Oral drugs are limited due to their undesirable effects on health, including cutaneous, gastrointestinal problems, hematological, hypoglycemic coma, and disturbance of liver and kidney functions.

The management of diabetes largely depends on pharmacological interventions such as insulin and oral hypoglycemic agents, including alpha‐glucosidase and alpha‐amylase inhibitors (Shah et al. 2023). However, these agents are often associated with adverse effects, including gastrointestinal discomfort, hypoglycemia, and weight gain, necessitating the search for safer and more effective alternatives (Rauf, Ibrahim, et al. 2024). The increasing interest in plant‐based medicines stems from their affordability, lower risk of side effects, and wide availability. Plants have long been recognized as a valuable source of bioactive compounds with hypoglycemic and antioxidant properties. Examples include plants rich in polyphenols, flavonoids, and alkaloids, which are known to improve glucose metabolism and mitigate oxidative stress (Jacob and Narendhirakannan 2019). Hyperglycemia‐induced overproduction of reactive oxygen species (ROS) leads to oxidative damage to pancreatic β‐cells, impaired insulin signaling, and the development of secondary complications such as diabetic nephropathy and retinopathy (Waheed et al. 2022). Moreover, chronic oxidative stress exacerbates inflammation by activating nuclear factor‐kappa B (NF‐κB) and other pro‐inflammatory pathways, further accelerating tissue damage (Sazdova et al. 2023). Natural antioxidants, particularly those derived from medicinal plants, have shown promise in mitigating these effects by scavenging free radicals and enhancing the activity of endogenous antioxidant enzymes such as superoxide dismutase (SOD) and glutathione peroxidase (GPx) (Alhasaniah et al. 2025; Shah et al. 2025).

*Oenothera laciniata* (family: Onagraceae), commonly known as cut‐leaf evening primrose, is a member of the genus *Oenothera*, which includes over 145 species of medicinal plants widely recognized for their therapeutic properties (Kaur et al. 2025). The genus is known for its pharmacologically active phytoconstituents, including phenolic acids, flavonoids, triterpenoids, and tannins, which exhibit diverse bioactivities (Munir et al. 2017). Despite extensive documentation, the anti‐diabetic potential of *O. laciniata* remains unexplored. The present study aimed to evaluate the antidiabetic potential of the crude ethanolic extract of *O. laciniata* (EOL) and its solvent fractions n‐hexane (NHOL), dichloromethane (DCMOL), ethyl acetate (EAOL), and aqueous (AQOL) through comprehensive phytochemical, biochemical, and pharmacological analyses.

## 2 Materials and Methods

### 2.1 Chemicals

All chemicals used in this study were analytical grades. Ethanol (CAS number: 64‐17‐5), methanol (CAS number: 67‐56‐1), *n‐*hexane (CAS number: 110‐54‐3), acetone (CAS number: 67‐64‐1), dichloromethane (CAS number: 75‐09‐2), ethyl acetate (CAS number: 141‐78‐6), ABTS [2,2′‐azino‐bis(3‐ethylbenzothiazoline‐6‐sulfonic acid)] (CAS number: 30931‐67‐0), DPPH (2,2‐diphenyl‐1‐picrylhydrazyl) (CAS number: 1898‐66‐4), acarbose (CAS number: 56180‐94‐0), ascorbic acid (CAS number: 67‐56‐1), hydrogen peroxide (CAS number: 7722‐84‐1), alloxan (CAS number: 2244‐11‐3), Tween 80 (CAS number: 9005‐65‐6), phosphate buffer (CAS number: 7778‐77‐0).

### 2.2 Plant Collection and Extraction

Specimens of *O. laciniata* were collected from District Swabi, Khyber Pakhtunkhwa, Pakistan, during the month of March 2022. The plant species was taxonomically identified and authenticated by Professor Dr. Naveed Akhtar, Department of Botany, Islamia College Peshawar, Pakistan. A voucher specimen (accession no. OL‐Bot‐15112022) was deposited in the Herbarium of the Department of Botany, University of Swabi, for future studies.

#### 2.2.1 Extraction Process

Upon collection, the fresh plant material was thoroughly rinsed with tap water to remove soil and other surface contaminants. The cleaned plant material was then air‐dried under shade for a period of 22 days to prevent photodegradation and loss of volatile constituents. Once fully dried, the plant material was ground into a fine powder using an electric grinder, yielding a total of 21 kg of powdered biomass. The powdered material was subjected to maceration using 150 L of 80% ethanol (v/v) for a period of 14 days, with occasional stirring to enhance extraction efficiency, as previously described (Khan, Pervaiz, et al. 2025). Following maceration, the solution was first filtered through muslin cloth and subsequently through Whatman No. 1 filter paper to remove plant residues. The combined filtrates were concentrated under reduced pressure using a rotary evaporator set at 40°C. To ensure complete removal of residual solvent, the concentrate was further dried using a water bath. This process yielded 443 g of crude ethanolic extract.

### 2.3 Fractionation

The crude ethanolic extract (EOL) was subjected to liquid–liquid partitioning to obtain solvent fractions of varying polarity. For this purpose, EOL was added in 500 mL of distilled water and transferred to a separatory funnel. An equal volume (500 mL) of n‐hexane (NHOL) was added, and the mixture was vigorously shaken to facilitate phase separation. After settling, the upper n‐hexane layer was collected. This extraction step was repeated three times, each with fresh 500 mL of n‐hexane. All n‐hexane fractions were pooled and concentrated under reduced pressure using a rotary evaporator to yield the n‐hexane fraction. The aqueous layer remaining after n‐hexane extraction was subsequently subjected to successive solvent partitioning with dichloromethane (DCMOL), ethyl acetate (EAOL), and n‐butanol, following the same protocol: three successive extractions with 500 mL of each solvent. Each organic fraction was separately collected, pooled, and concentrated using a rotary evaporator. The residual aqueous phase was retained as the aqueous fraction (AQOL). This sequential fractionation resulted in the isolation of five fractions: NHOL (83 g), DCMOL (417 g), EAOL (29 g), EOL (443 g) (residual crude), and AQOL (70 g) (Gul et al. 2025).

### 2.4 GC–MS Analysis

GC–MS analysis was performed using an Agilent 5975 GC–MSD system. The column used had a film thickness of 0.25 μm, a length of 30 m, and an internal diameter of 0.32 mm. Helium served as the carrier gas with a flow rate of 10 mL/min. The column's temperature was initially set at 80°C and gradually increased to 290°C at a rate of 10°C/min. The injector temperature was kept at 250°C with a split ratio of 1:100. A sample volume of 1 μL, dissolved in ethyl acetate, was injected into the system. Detection was carried out using a Mass Selective Detector. The relative percentage of each compound was calculated by dividing the average peak area of each compound by the total peak area of all identified compounds. The identities of the detected peaks were confirmed by comparing them with the data from the National Institute of Standards and Technology (NIST) library (Version 2.0, 2005), which provided the names and molecular weights of the sample components (Mahnashi, Alqahtani, et al. 2022).

### 2.5 Chemical and Pharmacological Assay

#### 2.5.1 In Vitro Antioxidant Assays

##### 2.5.1.1 DPPH Radical Scavenging Assay

DPPH scavenging activity was performed according to previously documented methods. 0.004% solution of DPPH was prepared freshly in methanol. Dilutions of various concentrations (500, 250, 125, 62.5, and 31.25 μg/mL) of the plant extract were prepared. Two microliter of 0.004% DPPH solution was added to 2 mL of each dilution of sample. The test tubes were kept in the dark for 30 min. A spectrophotometer model (Shimadzu UV‐1800) was used to measure the absorption at 517 nm while methanol served as blank. The assay was performed in triplicate (*n* = 3 independent experiments) using ascorbic acid as a standard (Sadiq et al. 2022). Percent inhibition was calculated by using the following formula:

$$\%\text{Radical scavenging activity}=\left[\left(A_{0}-A_{1}\right)/A_{0}\right]\times 100$$

where as *A*_0_ = control absorbance; *A*_1_ = sample absorbance.

##### 2.5.1.2 ABTS Free Radical Scavenging Assay

The antioxidant potential of samples was evaluated using ABTS free radical scavenging assay. Stock solution of 7 mM of ABTS and 2.45 mM of potassium per sulfate was prepared by mixing them in equal volumes 16 h before the assay. The stock solution was diluted with methanol to achieve absorbance of 0.7 units at wavelength of 734 nm using spectrophotometer (Sidiq et al. 2025). A fresh ABTS solution was prepared for each ABTS radical scavenging activity. Two microliter of ABTS solution was mixed with 2 mL of various strength solutions of the plant extract/fractions. The test tubes were kept in dark for 10 min and absorbance was calculated (Mahnashi et al. 2023). The assay was performed in triplicate (*n* = 3 independent experiments), and results were expressed as mean ± SEM. Ascorbic acid was used as standard. Percentage inhibition was calculated using the aformentioned formula.

##### 2.5.1.3 Hydrogen Peroxide Scavenging Assay

A solution of H_2_O_2_ (40 mM) was prepared in phosphate buffer (pH 7.4). Different concentrations (250 μL, 500 μL, 1000 μL/mL) of the plant extract and ascorbic acid as standard were added to a H_2_O_2_ solution (0.6 mL, 40 mM) . After 10 min of the absorbance of H_2_O_2_ solutions were calculated at 230 nm using spectrophotometer. Phosphate buffer was used as blank (Hussain et al. 2024; Masood et al. 2023). The assay was performed in triplicate (*n* = 3 independent experiments), and ascorbic acid was used as standard.

#### 2.5.2 In Vitro Anti‐Diabetic Activities

##### 2.5.2.1 In Vitro Assay of Alpha‐Glucosidase Activity

To measure alpha‐glucosidase activity for in vitro testing, samples were produced by adding 200 μL alpha‐glucosidase (0.5 mg/11 mL in d/H_2_O) was mixed with 200 μL glucopyranoside (15 mg/10 mL) in 1200 μL phosphate buffer solvent. This mixture acted as control. Samples were prepared by adding 200 μL glucopyranoside, 200 μL alpha‐glucosidase, and 1200 μL phosphate buffer solvent to 100 μL of different concentrations (31.25, 62.5, 125, 250, and 500 μg/mL) of the extract. The control and samples thus prepared were incubated at 37°C for 20 min. The reaction was stopped by pouring HCL after incubation of the mixture. Ultraviolet visible spectrophotometer was used to determine absorbance at 540 nm. Acarbose was used as standard in this assay (Alshehri et al. 2022). The assay was performed in triplicate (*n* = 3 independent experiments), and percentage inhibition was calculated using the following formula:

$$\text{Percent inhibition}=\frac{\text{Absorbance of control}-\text{Absorbance of sample}}{\text{Absorbance of control}}\times 100$$

##### 2.5.2.2 In Vitro Assay of Alpha‐Amylase Activity

The previously described methodology was used to study alpha‐amylase activity (Yousefi et al. 2013). Alpha‐amylase was mixed with phosphate buffer (pH 6.9), and different concentrations (31.25, 62.5, 125, 250, and 500 μg/mL) of the plant extracts were added to this solution. A 1% starch solution was also added to this mixture. The mixture was warmed by keeping it in a water bath for 5 min. Acarbose was used as standard. Ultraviolet‐visible spectrophotometer was used to record the color change at 656 nm. The assay was performed in triplicate (*n* = 3 independent experiments), and the percent inhibition was determined by the aforementioned formula.

#### 2.5.3 In Vivo/Animal Studies

Prior to the in vivo studies the ethical approval for all experimental animals was obtained from the ethical committee of Bacha Khan University, Charsadda Pakistan with approval number BKUC‐12/025. All the procedures were performed in accordance with institutional guidelines and internationally accepted guidelines and principles for the care and use of laboratory animals.

#### 2.5.4 Acute Toxicity Study

For toxicity studies of various fractions of the extract, the animals were divided into seven groups each consisting of five animals (*n* = 5 per group). The fractions were administered intraperitoneally at doses between 200 and 1500 mg/kg of the animal's body weight. Each animal was individually observed for signs of toxicity, behavioral changes, and mortality at regular intervals during the first 24 h and subsequently for up to 3 days post‐administration (Mahmood et al. 2019). Observations were recorded for each animal in all groups to ensure accurate assessment of toxic effects.

#### 2.5.5 In Vivo Activities

##### 2.5.5.1 Animals Studies

The experimental animals were maintained under standard laboratory conditions with a controlled environment (temperature 22°C ± 2°C, relative humidity 50%–60%, and a 12 h light/dark cycle) and were housed in polypropylene cages with free access to a standard pellet diet and water ad libitum, except during the pre‐induction fasting period. Prior to diabetes induction, animals were fasted for 12–36 h while allowing free access to water, as commonly recommended for alloxan‐based models to enhance diabetogenic efficacy. Diabetes mellitus was induced by a single intraperitoneal injection of freshly prepared alloxan monohydrate, and blood glucose levels were monitored at 48 and 72 h post‐administration. Animals exhibiting fasting blood glucose levels ≥ 200 mg/dL were considered diabetic and included in the study. This timeline for confirmation of hyperglycemia and the described housing and feeding regimen are consistent with established protocols reported in recent experimental studies employing alloxan‐induced diabetic models (Sunil et al. 2024).

##### 2.5.5.2 Experimental Design

A total of 90 animals were subject to experiments to assess the anti‐diabetic activity of various plant extracts. Animals were grouped into 8, each having five animals (*n* = 5 per group). Group I was declared as normal group and normal saline was administered only. Group II was designated as Diabetic group and Tween 80 was given. Group III (diabetic group) was administered standard drug glibenclamide (GB), whereas Group IV, Group V, Group VI, Group VII, and Group VIII were administered extracts of *O. laciniata* . The blood glucose of all animals was checked and recorded on the 0th, 5th, 10th, and 15th day of the plant extract administration.

#### 2.5.6 Biochemical Assays

After administering the extracts, blood samples were obtained from the slightly anesthetized animals' retro‐orbital plexus at the initial, first, second, third, and fourth hours as well as the initial, fifth, tenth, and fifteenth days after administration of extracts. In both the treated and control animals, changes were recorded in the blood levels of Serum Glutamic Pyruvic Transaminase (SGPT), Serum Glutamic Oxaloacetic Transaminase (SGOT), Alkaline Phosphatase (ALP), creatinine, and cholesterol (Mahnashi et al. 2023).

#### 2.5.7 Oral Glucose Tolerance Test (OGTT)

To evaluate oral glucose tolerance, baseline fasting blood glucose levels were recorded after overnight fasting. Glucose was then administered orally at a dose of 2 g/kg body weight. Blood glucose levels were measured at 0 (baseline), 30, 60, 90, and 120 min after glucose administration using blood samples collected from each animal. Each experimental group consisted of five animals (*n* = 5), and all glucose measurements were recorded for each individual animal at all time points (Mahnashi et al. 2023).

### 2.6 Histopathological Studies

After 1 days of treatment with the test extracts at the highest dose (300 mg/kg), the animals were anesthetized with ether, and the abdominal cavity was opened under aseptic conditions. The pancreas, liver, kidneys, and testes were carefully excised, rinsed in normal saline to remove blood and debris, and immediately fixed in 10% neutral buffered formalin for 24–48 h. Following fixation, tissues were dehydrated in a graded ethanol series, cleared in xylene, and embedded in paraffin wax. Thin sections of approximately 5 μm were prepared using a rotary microtome. The sections were mounted on glass slides, stained with hematoxylin and eosin (H&E), and examined under a light microscope for histopathological alterations (Imtiaz et al. 2023).

### 2.7 Docking Studies

Molecular docking is a cornerstone of structure‐based drug discovery, providing predictive insights into the binding behavior of novel drug candidates with their biological targets (Mahnashi et al. 2024). Docking studies computationally model ligand‐target interactions, enabling the estimation of binding affinity, identification of key intermolecular interactions, and rational optimization of lead compounds. This approach of performing the research studies can reduce the experimental cost and time by prioritizing the promising candidates for synthesis and biological evaluations. Through this approach, a number of changes can be given to the synthesized compound as well as the biological target before utilizing the other experimental approaches (Ghazi et al. 2018). In this research work, GC–MS analysis components of different solvent fractions of *O. laciniata* were extracted and their pharmacological activities were analyzed. The structures of targeted components were drawn through Chem Draw 8.0 and saved in pdb format. Meanwhile, the macromolecular structures of targeted proteins were downloaded from an online protein data server, that is, www.rcsb.com and saved in pdb format. The structures of Glycosidase were obtained as Pdb ID: 2ZEO and alpha amylase as pdb ID: 3BC9. These structures were further modified and purified through BioVia Discovery Studio Visualizer and resaved in pdb format. These all targeted proteins and ligand structures were reopened in docking software PyRx interlinked with Auto Dock Vina and saved in pdbqt format. This format is considered good to perform the docking studies. Parameters were set and energy was minimized along with specific selection of grid around the active site that increases the probability of attaching to the proteins. Docking was performed and results were saved. Those postures were selected that gave increased negative binding energies. The best postures were reopened in Discovery Studio Visualizer and images were drawn through the same software and Pymol that gave advanced insights of results.

### 2.8 Data Analysis

Different statistical software's such as graph pad prism 5.1 and excel was used for analysis of data. Data were analyzed as mean ± SEM, two‐way ANOVA followed by Bonferroni test was followed for the statistical analysis. ***p < 0.001, **p < 0.01, *p < 0.05, ns, not significant (*p* > 0.05).

## 3 Results and Discussion

Diabetes mellitus is a long‐term metabolic disorder that is either caused by a malfunctioning insulin secretory system or by insulin resistance or both (Alshehri et al. 2022). In addition to disruptions in glucose metabolism, DM is now well known as a multifactorial disease having complex biochemical and molecular changes that lead to the development of long‐term complications. These complications, such as neuropathy, nephropathy, retinopathy, and cardiovascular diseases, have a significant impact on morbidity and mortality in diabetic patients (Rauf, Almasoud, et al. 2024). Oxidative stress is a major underlying cause of the development of diabetes and its related complications. In hyperglycemic states, excessive glucose flux stimulates a variety of metabolic pathways, such as the polyol pathway, activation of protein kinase C, and the production of advanced glycation end products (AGEs) (Alshehri et al. 2024). Of them, AGEs play a key role by interacting with their receptors (RAGE), leading to the activation of intracellular signaling cascades and stimulation of NADPH oxidase, which significantly increases the production of reactive oxygen species (ROS) (Alkadi 2020). Moreover, electron leakage and superoxide production as a result of mitochondrial dysfunction caused by chronic hyperglycemia are also contributing factors to oxidative stress. Excessive production of ROS alters cellular homeostasis and causes oxidative damage to lipids, proteins, and nucleic acids. Oxidative damage of pancreatic β‐cells is especially vulnerable to oxidative damage because of their intrinsically low capacity to defend against oxidative damage. In addition, ROS disrupts the insulin signaling pathways; thus, finding its way into insulin resistance (Hussain et al. 2024). In line with the earlier findings, diabetic conditions are also characterized by a reduction in the levels of endogenous antioxidants like reduced glutathione (GSH) and vitamin C, as well as altered activities of key antioxidant enzymes, such as superoxide dismutase (SOD) and catalase (CAT). This imbalance enhances lipid peroxidation and membrane damage, which ultimately promotes endothelial dysfunction and atherosclerosis. Notably, oxidative stress is one of the leading contributors to the occurrence of diabetic complications (Khan et al. 2022). Higher levels of ROS trigger inflammatory processes, microvascular damage, and apoptosis, which are the key processes in the pathogenesis of diabetic neuropathy, retinopathy, nephropathy, and cardiovascular disorders. Nutritionally speaking, this underscores the importance of dietary antioxidants in counteracting the effects of oxidative stress and enhancing the metabolic performance of diabetic patients (Mahnashi, Alyami, et al. 2022). Naturally occurring bioactive compounds, such as polyphenols, flavonoids, and heterocyclic compounds, have been demonstrated to have significant antioxidant and antidiabetic potential by scavenging and depleting free radicals, improving endogenous antioxidant defenses, and modulating key metabolic pathways (Ratan et al. 2024).

### 3.1 Phytochemical Composition of DCMOL

The GC–MS analysis of the DCM fraction of *O. laciniata* revealed eight major constituents. The Bis(2‐ethylhexyl) phthalate was the predominant compound (62.48%), a plasticizer and has been also reported in some other plant like *Withania somnifera* and *Azadirachta indica* (Nema and Khare 2020). The second most abundant compound was 2,2,4‐trimethyl‐1,3‐pentanediol di‐isobutyrate (15.14%), another plasticizer associated with industrial exposure (Tiwana et al. 2025). Linoleic acid (9.91%), a bioactive omega‐6 fatty acid with known antioxidant and anti‐inflammatory was also detected (Tortosa‐Caparrós et al. 2017). Additionally, Ethylbenzene (7.55%) and p‐xylene (4.41%), both recognized environmental pollutants, were identified suggesting possible uptake from surrounding air or soil (Mirzaei et al. 2022). The Phenol, 2,4‐bis(1,1‐dimethylethyl)‐ (3.37%), a synthetic antioxidant (BHT derivative), was present in moderate quantity. Minor constituent included palmitic acid (2.38%), a fatty acid reported antimicrobial and antioxidant effects (Danesh et al. 2021). The mcrocyclic lactone 13‐Hexyloxacyclotridec‐10‐en‐2‐one (2.25%) and industrial solvent cyclohexanone (2.01%) an industrial solvent. Overall the presence of several plasticizer and pollutants indicates potential environmental contamination infusing the chemical profile of DCM fraction as shown in Table 1 and Figure S1.

**TABLE 1:** GC–MS analysis of DCM fractions of *Oenothera laciniata*.

ID#	Name	R‐time (min)	Area	Conc. (%)	Molecular weight (g/mol)	Molecular formula
1	p‐Xylene	2.683	7,945,391	4.41	106.165	C_8_H_10_
2	Cyclohexanone	2.752	3,617,030	2.01	98.15	C_6_H_10_O
3	Phenol, 2,4‐bis (1,1‐dimethylethyl)‐	12.459	6,068,857	3.37	198.28	C_16_H_22_O_2_
4	2,2,4‐Trimethyl‐1,3‐pentanediol diisobutyrate	13.449	9,272,203	15.14	290.44	C_14_H_20_O_4_
5	n‐Hexadecanoic acid	17.391	5,182,542	2.38	256.42	C_16_H_32_O_2_
6	13‐Hexyloxacyclotridec‐10‐en‐2‐one	18.275	4,057,543	2.25	238.36	C_13_H_26_O_2_
7	9, 12‐Octadecadienoic acid (Z, Z)‐	19.003	17,867,749	9.91	280.44	C_18_H_32_O_2_
8	Bis (2‐ethylhexyl) phthalate	22.205	112,609,189	62.48	390.56	C_24_H_38_O_4_

#### 3.1.1 Phytochemical Composition of EAOL

The present results are similar to the chemical compositions of *Acmella oleracea* and *Achillea millefolium* , which have been reported for antimicrobial activity (Gilbert et al. 2022). Comparative phytochemical studies in *O. biennis* disclosed comparable contents of fatty acids and polyphenols (gallic acid, quercetin, and catechin), reinforcing the phytochemistry conservation within the *Oenothera* genus. *O. laciniata's* anti‐inflammatory activity is consistent with earlier findings of inhibitory NO and cytokine synthesis in macrophages due to polyunsaturated fatty acids and phytosterols (Yoon et al. 2009).

The results show 19 compounds in ethyl acetate of plant sample through GC–MS (Table 2 and Figure S2). The highest concentration compounds are octadecanoic acid (23.76%), 9,12‐octadecadienoic acid (Z,Z)‐ (15.22%), 9,12‐octadecadienoic acid (Z,Z)‐ (13.14%), 9,12‐octadecadienoic acid (Z,Z)‐ (8.90%), (R)‐(‐)‐14‐methyl‐8‐hexadecyn‐1‐ol (7.19%), γ‐sitosterol (4.95%), propanoic acid ester (4.57%), n‐hexadecanoic acid (4.57%), 9,12‐octadecadienoic acid (Z,Z)‐ (3.77%), n‐hexadecanoic acid (3.36%), 9,12‐octadecadienoic acid (Z,Z)‐ (3.44%), 9,12‐octadecadienoic acid (Z,Z)‐ (2.00%), butyl 9,12‐octadecadienoate (1.68%), and benzoic acid (1.34%), respectively. The ethyl acetate fraction isolated 19 compounds, dominated by octadecanoic acid (23.76%), 9,12‐octadecadienoic acid (Z, Z)‐ (15.22%), and γ‐sitosterol (4.95%).

**TABLE 2:** GC–MS analysis of EAOL fractions of *Oenothera laciniata*.

ID#	Name	R‐time (min)	Area	Conc. (%)	Molecular weight (g/mol)	Molecular formula
1	1,2,3‐Propanetriol, 1‐acetate	4.636	24,503,901	1.16	134.13	C_5_H_10_O_4_
2	Benzoic acid	7.873	28,377,939	1.34	122.12	C_6_H_6_O
3	2(3H)‐Furanone, 5‐hexyldihydro‐	7.873	28,377,939	1.34	154.21	C_8_H_12_O_2_
4	Propanoic acid, 2‐methyl‐, 1‐(1,1‐dimethylethyl)‐2‐methyl‐1,3‐propanediyl ester	13.441	96,712,872	4.57	286.41	C_16_H_30_O_4_
5	n‐Hexadecanoic acid	13.441	96,712,872	4.57	256.42	C_16_H_32_O_2_
6	n‐Hexadecanoic acid	17.23	71,171,184	3.36	256.42	C_16_H_32_O_2_
7	n‐Hexadecanoic acid	17.337	14,095,994	0.67	256.42	C_16_H_32_O_2_
8	9, 12‐Octadecadienoic acid (Z, Z)‐	17.379	42,245,174	2	280.45	C_18_H_32_O_2_
9	9, 12‐Octadecadienoic acid (Z, Z)‐	18.932	278,121,182	13.14	280.45	C_18_H_32_O_2_
10	Octadecanoic acid	19.012	502,988,701	23.76	284.48	C_18_H_36_O_2_
11	9, 12‐Octadecadienoic acid (Z, Z)‐	19.22	72,873,618	3.44	280.45	C_18_H_32_O_2_
12	9, 12‐Octadecadienoic acid (Z, Z)‐	19.349	322,326,831	15.22	280.45	C_18_H_32_O_2_
13	9, 12‐Octadecadienoic acid (Z, Z)‐	19.548	79,905,552	3.77	280.45	C_18_H_32_O_2_
14	9, 12‐Octadecadienoic acid (Z, Z)‐	19.697	188,414,164	8.9	280.45	C_18_H_32_O_2_
15	cis‐13‐Eicosenoic acid	20.532	28,636,897	1.35	310.51	C_20_H_38_O_2_
16	Butyl 9,12‐octadecadienoate	20.704	35,523,401	1.68	336.55	C_22_H_40_O_2_
17	Bis(2‐ethylhexyl) phthalate	21.605	7,930,980	0.37	390.56	C_24_H_38_O_4_
18	(R)‐(−)‐14‐Methyl‐8‐hexadecyn‐1‐ol	22.201	152,245,501	7.19	266.47	C_17_H_36_O
19	γ‐Sitosterol	32.315	104,838,099	4.95	414.74	C_29_H_50_O_2_

#### 3.1.2 Phytochemical Composition of NHOL

The n‐hexane fraction produced fourteen compounds, with γ‐sitosterol (25.80%), 9,12‐octadecadienoic acid (18.87%), and palmitic acid (8.41%) as the major constituents. The fatty acids and phytosterols are of pharmaceutical significance, displaying antioxidant, anti‐inflammatory, and antidiabetic activities (Al‐Snafi 2020). Linoleic acid, which is rich in both extracts, contributes to anti‐inflammatory signaling and insulin sensitivity (Koba and Yanagita 2014). Comparable compositions in *O. biennis* connect linoleic acid with oxidative stress inhibition (Asadi‐Kavan et al. 2020). γ‐Sitosterol, which predominates in the n‐hexane extract, relates to antioxidant and glucose‐modulating activities, consistent with findings in *Leucas asperaand O. speciosa* (Goyal et al. 2022). Vitamin E (α‐tocopherol) and phytol were also found, with confirmation of antioxidant activity. Vitamin E neutralizes free radicals and guards against lipid peroxidation of membranes, and phytol provides anti‐inflammatory and anticancer activities. *O. paradoxa* has similar activities from compounds (Gorlach et al. 2011). The other identified constituents, like methyl stearate, benzoic acid, and propanoic acid esters, are linked with antimicrobial activities. Benzoic acid, for instance, is an antimicrobial of broad application as a natural preservative. The NHOL fraction detected fourteen compounds via GC–MS mentioned in (Table 3 and Figure S3). The most abundant compounds found in the extract of *O. laciniata* , as per their relative contents, are gamma‐Sitosterol (25.80%), 9,12‐Octadecadienoic acid (Z,Z)‐ (18.87%), Methyl 9‐cis,11‐trans‐octadecadienoate (9.09%), n‐Hexadecanoic acid (8.41%), Pentadecanoic acid, 14‐methyl‐, methyl ester (6.10%), 9,12,15‐Octadecatrienoic acid, methyl ester (Z,Z,Z)‐ (6.05%), 9‐Octadecenoic acid, methyl ester (E)‐ (2.30%), Bis(2‐ethylhexyl) phthalate (2.20%), Hexadecanoic acid, 2‐hydroxy‐1‐(hydroxymethyl)ethyl ester (2.71%), Vitamin E (2.71%), Phytol (1.32%), O‐Xylene (1.21%), Methyl stearate (0.89%), and Octadecanoic acid (0.81%). These constituents, which are primarily composed of fatty acid esters, phytosterols, and antioxidants, are largely responsible for the pharmacological activities of the plant, such as its antidiabetic and antioxidant activity.

**TABLE 3:** GC–MS analysis of NHOL fractions of *Oenothera laciniata*.

ID#	Name	R‐time	Area	Conc. (%)	Mol. weight (g/mol)	Mol. formula
1	Cyclohexane, ethyl‐	2.363	13,019,253	0.31	112.21	C_8_H_16_
2	O‐Xylene	2.667	49,864,511	1.21	106.17	C_8_H_10_
3	P‐Xylene	2.974	15,346,557	0.37	106.17	C_8_H_10_
4	Nonane	3.25	17,131,282	0.41	128.26	C_9_H_20_
5	2(3H)‐Furanone, 5‐hexyldihydro‐	11.573	23,817,646	0.58	156.23	C_10_H_16_O_2_
6	2‐Pentadecanone, 6,10,14‐trimethyl‐	16.19	14,628,080	0.35	268.48	C_18_H_36_O
7	Pentadecanoic acid, 14‐methyl‐, methyl ester	16.996	252,400,831	6.1	270.46	C_17_H_34_O_2_
8	n‐Hexadecanoic acid	17.443	347,803,470	8.41	256.42	C_16_H_32_O_2_
9	Methyl 9‐cis,11‐trans‐octadecadienoate	18.56	376,239,595	9.09	294.46	C_19_H_34_O_2_
10	9,12,15‐Octadecatrienoic acid, methyl ester (Z,Z,Z)‐	18.595	250,292,986	6.05	292.45	C_19_H_32_O_2_
11	9‐Octadecenoic acid, methyl ester (E)‐	18.631	94,993,629	2.3	296.49	C_19_H_36_O_2_
12	Phytol	18.802	54,607,317	1.32	296.53	C_20_H_40_O
13	Methyl stearate	18.89	36,779,623	0.89	298.5	C_19_H_38_O_2_
14	9, 12‐Octadecadienoic acid (Z, Z)‐	19.026	780,750,831	18.87	280.45	C_18_H_32_O_2_
15	Octadecanoic acid	19.263	33,650,425	0.81	284.48	C_18_H_36_O_2_
16	Isopropyl linoleate	21.635	28,253,428	0.68	308.51	C_21_H_38_O_2_
17	Hexadecanoic acid, 2‐hydroxy‐1‐(hydroxymethyl)ethyl ester	21.952	112,190,670	2.71	330.5	C_19_H_38_O_4_
18	Bis (2‐ethylhexyl) phthalate	22.207	91,047,224	2.2	390.56	C_24_H_38_O_4_
19	Vitamin E	28.646	112,108,472	2.71	430.71	C_29_H_50_O_2_
20	gamma‐Sitosterol	32.451	1,067,592,577	25.8	414.71	C_29_H_50_O

### 3.2 Antioxidant Activity of *O. laciniata* Extracts

#### 3.2.1 ABTS, DPPH, and H_2_O_2_ Radical Scavenging Activity

The antioxidant potential of the plant extracts was evaluated using ABTS, DPPH, and H_2_O_2_ scavenging assays. ABTS and DPPH assays were selected for investigation of antioxidant activity because of their widespread use, speed of results, sensitivity, and use of stable species (Hussain 2020). DCM extract exhibited the highest antioxidant activity across all assays, as evidenced by its lowest IC_50_ values: 3.64 μg/mL for ABTS (*p* < 0.001), 5.38 μg/mL for DPPH (*p* < 0.001), and 21.60 μg/mL for H_2_O_2_ (*p* < 0.001). The GC–MS spectrum of the DCMOL fraction showed the presence of important antioxidants like Phenol, 2,4‐bis(1,1‐dimethylethyl), n‐hexadecanoic acid, and 9,12‐octadecadienoic acid (linoleic acid). These compounds have been found to be effective in scavenging free radicals and inhibiting lipid peroxidation (Christopher et al. 2022). More interestingly, the presence of phenolic derivatives in the DCMOL fraction is very convincing evidence of its higher ability to scavenge ABTS and DPPH radicals since phenolic compounds function as hydrogen/electron donors. In addition, the higher activity of the DCMOL fraction (IC_50_ = 3.64 μg/mL in ABTS) as an antioxidant than that of ascorbic acid may indicate some synergistic effects between the different active fractions within the extract, a common scenario for most plant extracts. The EAOL and NHOL extracts also showed significant but lower antioxidant activity as compared to the DCMOL fraction.

The GC–MS results of the EAOL fraction revealed a significant presence of fatty acids including octadecanoic acid and 9,12‐octadecadienoic acid, along with other bioactive molecules such as γ‐sitosterol. Although these molecules exhibit antioxidant and anti‐inflammatory activity, their mode of action is relatively weaker in terms of radical scavenging than that of the phenolic compounds (Eisa et al. 2026), thus the elevated IC_50_ values of the EA fraction. Similarly, the NHOL revealed lower activity. The decreased activity can thus be explained by the higher proportion of non‐polar substances such as hydrocarbons and fatty acid esters. Such substances are less active in terms of donating hydrogen ions in ABTS and DPPH tests. However, the presence of the strong antioxidant γ‐sitosterol (25.8%) and vitamin E in NHOL did not affect antioxidant activity (Hsu et al. 2017) in the experiments as much as it could have due to their different solubilities and other characteristics of in vitro tests. Antioxidant activity clearly correlated with solvent polarity, as well as with the efficiency of extracting compounds from the sample. Medium‐polarity substances, such as dichloromethane, can better extract antioxidants than low‐polarity substances, such as n‐hexane (Widyawati et al. 2014). As a rule, low‐polarity substances tend to extract lipids, which possess rather low antioxidant properties compared to phenols. These findings underscore the potential of *O. laciniata* extracts as natural antioxidants that could mitigate oxidative stress and its associated complications in DM (Kwak et al. 2019).

**FIGURE 1:**
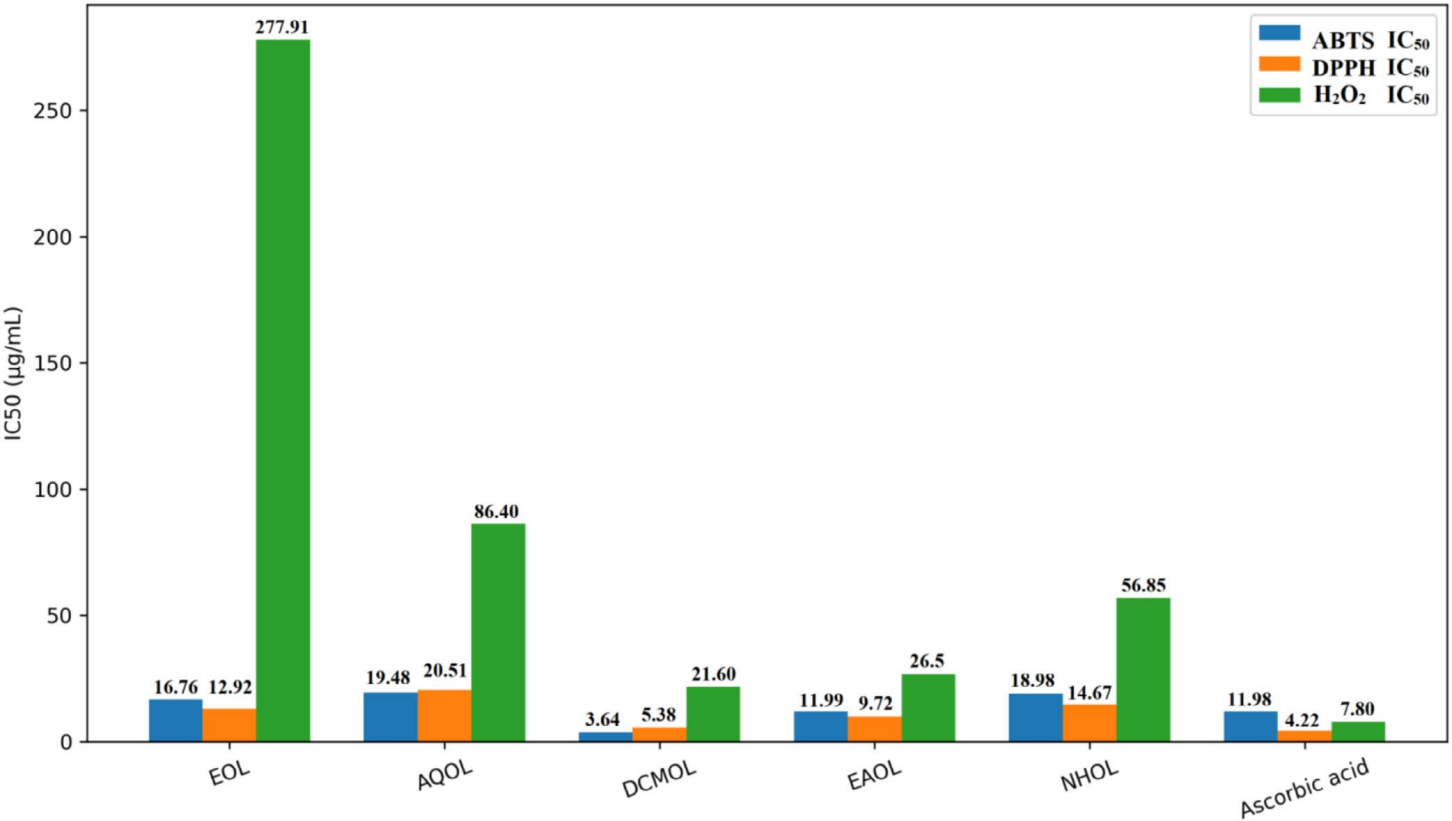
IC_50_ values of *Oenothera laciniata* extract fractions assessed via ABTS, DPPH, and H_2_O_2_ free radical scavenging assays.

The maximum ABTS % inhibition was recorded by ascorbic acid (94.08% ± 1.04%) at 500 μg/mL, closely followed by dichloromethane extract (89.88% ± 0.89%) and EAOL extract (88.61% ± 0.43%). Among extracts, DCMOL showed the lowest IC_50_ (3.64 μg/mL), much lower than the control ascorbic acid (11.98 μg/mL), pointing to higher ABTS radical scavenging activity. EOL and aqueous extracts were moderately active (IC_50_ = 16.76 and 19.48 μg/mL, respectively), whereas NHOL had the lowest ABTS activity among the extracts (IC_50_ = 18.98 μg/mL). Followed by DPPH Ascorbic acid showed maximum inhibition (97.23% ± 0.82%) and the minimum IC_50_ value (4.22 μg/mL). DCMOL extract was second with strong inhibition (87.45% ± 0.59%) and a low IC_50_ value of 5.38 μg/mL, thus exhibiting strong antioxidant activity. EAOL extract also demonstrated noteworthy activity (91.36% ± 1.49%, IC_50_ = 9.72 μg/mL), performing better than both EOL (IC_50_ = 12.92 μg/mL) and NHOL (IC_50_ = 14.67 μg/mL). AQOL extract revealed the poorest DPPH radical scavenging activity (IC_50_ = 20.51 μg/mL) among all the samples. For H_2_O_2_ scavenging activity the most potent was demonstrated with ascorbic acid (94.40% ± 0.03%, IC_50_ = 7.80 μg/mL). Among the plant extracts, DCMOL extract also recorded better activity (81.76% ± 0.71%, IC_50_ = 21.60 μg/mL), followed by EAOL (86.58% ± 1.12%, IC_50_ = 26.50 μg/mL). EOL and AQOL extracts recorded lesser H_2_O_2_ scavenging with IC_50_ values of 277.91 and 86.40 μg/mL, respectively. The least active extract was NHOL (IC_50_ = 56.85 μg/mL), but it still registered significant inhibition at higher doses (Table 4, Figure 1).

**TABLE 4:** Antioxidant potential of *Oenothera laciniata* extract fractions assessed via ABTS, DPPH, and H_2_O_2_ free radical scavenging assays.

Sample	Concentration (μg/mL)	ABTS % inhibition	DPPH % inhibition	H_2_O_2_% inhibition
EOL	500	85.32 ± 2.87***	77.40 ± 1.51***	67.73 ± 0.03***
	250	77.12 ± 0.54***	72.57 ± 3.84***	57.42 ± 0.12***
	125	72.79 ± 1.08***	67.36 ± 0.55***	47.39 ± 0.35***
	62.5	65.79 ± 1.88***	62.56 ± 0.95***	41.36 ± 0.71***
	31.25	57.20 ± 0.47***	57.37 ± 1.10***	29.15 ± 0.22***
AQOL	500	83.51 ± 0.54**	81.30 ± 1.42**	67.51 ± 0.54***
	250	75.76 ± 1.61***	75.78 ± 0.45**	62.84 ± 0.30***
	125	67.22 ± 1.28***	69.44 ± 0.86*	57.80 ± 1.50***
	62.5	63.51 ± 0.54***	62.72 ± 1.89**	52.72 ± 1.01***
	31.25	56.37 ± 2.56***	54.29 ± 2.64**	47.61 ± 0.43***
DCMOL	500	89.88 ± 0.89^ns^	87.45 ± 0.59**	81.76 ± 0.71***
	250	84.54 ± 3.60***	82.49 ± 0.60***	76.23 ± 1.83***
	125	79.01 ± 1.97***	77.23 ± 0.44***	71.42 ± 0.43***
	62.5	74.68 ± 0.22***	72.50 ± 0.61***	65.56 ± 1.06***
	31.25	71.82 ± 1.95***	67.47 ± 0.46***	63.80 ± 1.50***
EAOL	500	88.61 ± 0.43***	91.36 ± 1.49^ns^	86.58 ± 1.12***
	250	82.58 ± 0.63***	85.34 ± 0.55^ns^	81.65 ± 1.34***
	125	75.10 ± 0.60***	81.39 ± 2.49**	75.31 ± 2.15***
	62.5	69.25 ± 1.40***	76.47 ± 0.52***	68.56 ± 1.73***
	31.25	62.87 ± 0.85***	71.44 ± 2.55***	63.44 ± 0.58***
NHOL	500	79.37 ± 1.04***	88.52 ± 2.06**	71.60 ± 1.63***
	250	73.37 ± 0.54***	82.48 ± 0.60**	66.8 ± 70.85***
	125	69.30 ± 2.61***	74.54 ± 1.46**	61.25 ± 1.40***
	62.5	63.42 ± 1.05***	67.34 ± 2.63***	55.10 ± 0.60***
	31.25	53.52 ± 2.52***	61.30 ± 1.49***	51.61 ± 0.43***
Ascorbic acid	500	94.08 ± 1.04	97.23 ± 0.82	94.40 ± 0.03
	250	87.45 ± 0.90	92.45 ± 0.90	85.03 ± 2.16
	125	81.58 ± 2.63	85.90 ± 0.60	80.90 ± 1.11
	62.5	76.40 ± 3.20	81.00 ± 3.30	76.44 ± 0.28
	31.25	71.80 ± 0.90	76.90 ± 1.45	71.22 ± 0.47

*Note:* Values are expressed as mean ± SEM (*n* = 3). Statistical analysis was performed using two‐way ANOVA followed by Tukey's post hoc test. Extract type and concentration were treated as fixed factors, whereas experimental replicates were considered random effects. Statistical significance is indicated as: ns = non‐significant (*p* > 0.05), *p ≤ 0.05, **p ≤ 0.01, *p ≤ 0.001, compared with the standard (ascorbic acid). The interaction between extract type and concentration was found to be statistically significant (*p* < 0.001).

### 3.3 In Vitro Anti‐Diabetic Activities of *O. laciniata* Extracts

#### 3.3.1 Alpha‐Glucosidase and Alpha‐Amylase Enzyme Inhibition

α‐Glucosidase enzymes present in the small intestine are involved in the conversion of complex carbohydrates into glucose (Pervaiz et al. 2022). Hydrolysis of starch into glucose leads to increased postprandial blood glucose levels. Inhibition of these enzymes delays carbohydrate digestion and reduces postprandial hyperglycemia (Ullah et al. 2025). Clinically used α‐glucosidase inhibitors such as acarbose and miglitol act through this mechanism and are effective in managing diabetes mellitus (DM), thereby reducing the risk of complications including neuropathy, nephropathy, and retinopathy (Alshehri et al. 2022). Similarly, α‐amylase catalyzes the breakdown of starch into oligosaccharides, which are further converted into glucose by α‐glucosidase enzymes (Waheed et al. 2022). Therefore, inhibition of α‐amylase also contributes to improved glycemic control by delaying carbohydrate digestion and glucose absorption (Mahnashi et al. 2023).

**FIGURE 2:**
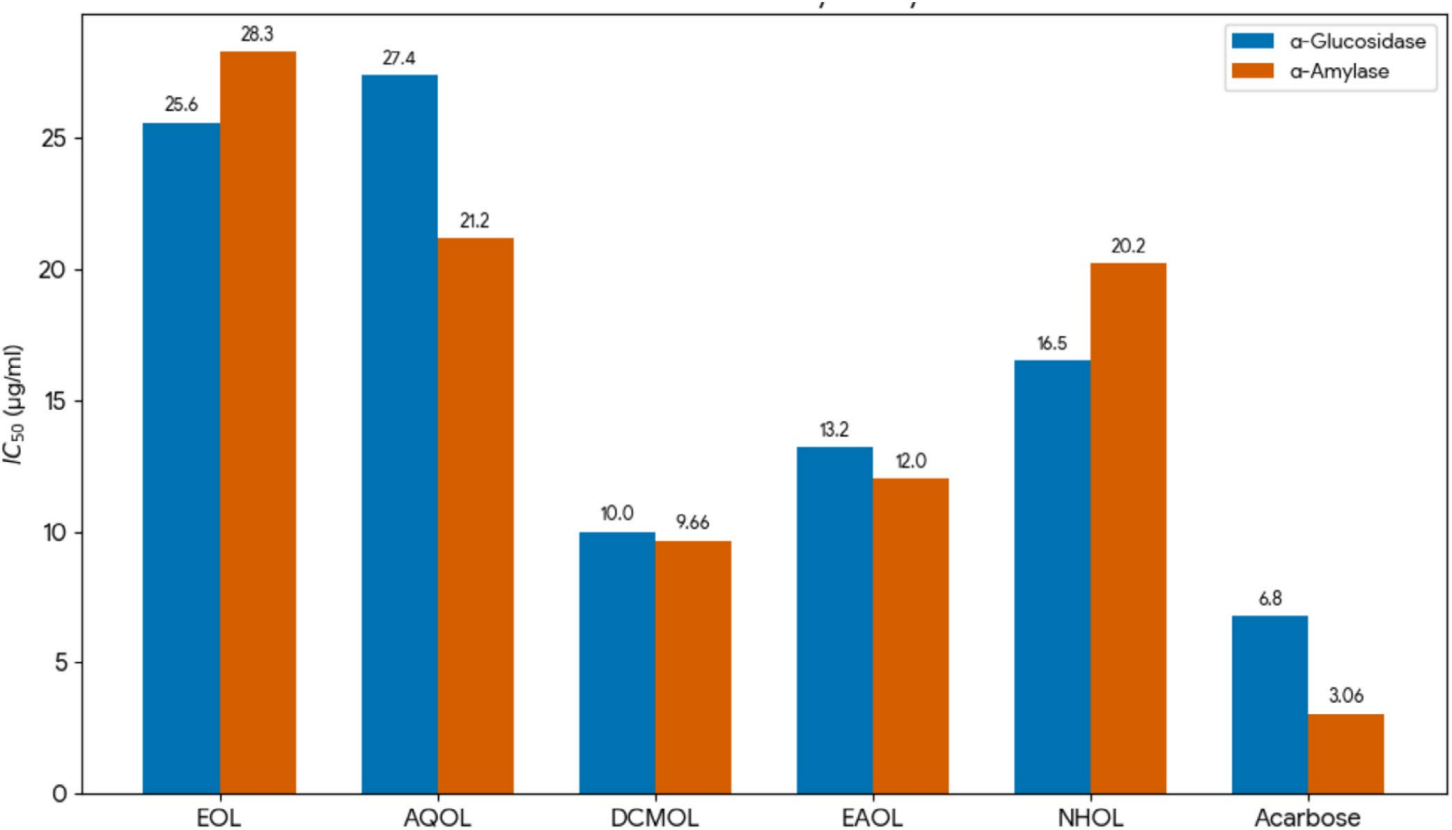
IC_50_ values of various fractions of *Oenothera laciniata* extracts assessed via α‐glucosidase and α‐amylase assay.

In the present study, all tested extracts exhibited significant dose‐dependent inhibition (*p* < 0.001) against both α‐glucosidase and α‐amylase enzymes. Among the fractions, dichloromethane (DCM), ethyl acetate (EA), and n‐hexane showed the most potent activity, whereas aqueous and crude extracts showed comparatively moderate effects. DCM fraction demonstrated the strongest inhibitory activity with IC_50_ values of 10 μg/mL (α‐glucosidase) and 9.66 μg/mL (α‐amylase), followed by EA (13.2 and 12 μg/mL) and n‐hexane (16.5 and 20.2 μg/mL), respectively. These values were significant when compared with acarbose, which showed IC_50_ values of 6.80 μg/mL (α‐glucosidase) and 3.06 μg/mL (α‐amylase). At 500 μg/mL, all extracts showed strong enzyme inhibition ranging from approximately 70% to 92%, depending on fraction and enzyme type. The crude extract (EOL) showed inhibition of 81.53% ± 0.71% (α‐glucosidase) and 88.58% ± 1.12% (α‐amylase), with IC_50_ values of 25.6 and 28.3 μg/mL, respectively. The aqueous fraction (AQOL) showed IC_50_ values of 27.4 μg/mL (α‐glucosidase) and 21.20 μg/mL (α‐amylase), indicating comparatively stronger α‐amylase inhibition. Among all fractions, DCMOL exhibited the highest potency, with maximum inhibition of 88.43% ± 1.26% (α‐glucosidase) and 92.67% ± 1.30% (α‐amylase) at 500 μg/mL, and IC_50_ values of 10 and 9.66 μg/mL, respectively. The EAOL also showed strong inhibitory activity with IC_50_ values of 13.2 and 12 μg/mL, whereas NHOL showed moderate activity with IC_50_ values of 16.5 and 20.2 μg/mL, respectively. Standard acarbose exhibited the strongest inhibition overall with consistently higher percentage inhibition across all concentrations as shown in Table 5 and Figure 2.

**TABLE 5:** In vitro antidiabetic activity of various fractions of *Oenothera laciniata* extracts.

Compound name	Dose (μg/mL)	% Alpha‐glucosidase inhibition	% Alpha‐amylase inhibition
EOL	500	81.53 ± 0.71***	88.58 ± 1.12**
	250	76.58 ± 1.12***	81.65 ± 1.34**
	125	71.42 ± 0.43***	74.31 ± 2.15**
	62.5	65.08 ± 0.47***	67.56 ± 1.73***
	31.25	62.72 ± 1.01***	62.44 ± 0.58***
AQOL	500	79.48 ± 0.74***	79.37 ± 0.69***
	250	75.62 ± 0.40***	74.72 ± 0.51***
	125	70.60 ± 0.46***	70.47 ± 0.59***
	62.5	57.68 ± 0.49***	65.50 ± 0.71***
	31.25	51.72 ± 0.66***	48.46 ± 0.72***
DCMOL	500	88.43 ± 1.26***	92.67 ± 1.30^ns^
	250	83.83 ± 0.66^ns^	88.58 ± 0.47^ns^
	125	77.93 ± 0.90^ns^	82.54 ± 0.68^ns^
	62.5	72.26 ± 0.77*	78.20 ± 1.24^ns^
	31.25	67.10 ± 0.95**	73.40 ± 0.42^ns^
EAOL	500	86.44 ± 0.58**	82.33 ± 1.20***
	250	81.08 ± 0.47*	76.33 ± 0.95***
	125	79.84 ± 0.30^ns^	72.67 ± 0.91***
	62.5	74.94 ± 1.13^ns^	70.00 ± 0.17**
	31.25	52.58 ± 0.63***	68.60 ± 0.04^ns^
NHOL	500	79.49 ± 0.60***	82.61 ± 0.77***
	250	75.58 ± 0.63***	77.60 ± 0.80***
	125	72.93 ± 0.67**	72.83 ± 0.56**
	62.5	65.44 ± 0.58***	57.55 ± 0.77***
	31.25	54.56 ± 1.73***	54.58 ± 0.74***
Acarbose	500	94.40 ± 0.03	93.08 ± 1.04
	250	85.03 ± 2.16	86.45 ± 0.90
	125	80.90 ± 1.11	80.58 ± 0.63
	62.5	76.44 ± 0.28	75.40 ± 0.20
	31.25	71.22 ± 0.47	70.80 ± 0.90

*Note:* Data are expressed as mean ± SEM (*n* = 3). Statistical analysis was performed using two‐way ANOVA with extract type and concentration treated as fixed factors, followed by Tukey's post hoc test. Interaction between factors was also assessed. Statistical significance is indicated as: ***p < 0.001, **p < 0.01, *p < 0.05, ns = not significant (*p* > 0.05), compared with standard (acarbose).

Overall, these findings suggest that the observed enzyme inhibitory activity may be attributed to bioactive phytochemicals such as flavonoids and phenolic compounds, which may interact with catalytic sites of α‐glucosidase and α‐amylase, thereby reducing carbohydrate hydrolysis and postprandial glucose elevation.

### 3.4 In Vivo Anti‐Diabetic Activities of *O. laciniata* Extracts Fractions in Diabetic Rats Induced by Alloxan

The in vivo anti‐diabetic activity of *O. laciniata* extract fractions demonstrated a significant dose‐dependent reduction in blood glucose levels in alloxan‐induced diabetic rats over a 15‐day treatment period, thereby confirming their potential antihyperglycemic effects. Among all tested fractions, DCMOL exhibited the most pronounced activity, reducing blood glucose to a mean value of 115.92 mg/dL and producing reductions of 207, 202, and 225 mg/dL at doses of 75, 150, and 300 mg/kg, respectively (*p* < 0.001). EAOL also showed strong antihyperglycemic activity, with reductions of 135, 139, and 207 mg/dL, whereas the n‐hexane fraction (NHOL) produced moderate effects with decreases of 56, 63, and 158 mg/dL at corresponding doses. AQOL and EOL exhibited comparatively weaker but still significant glucose‐lowering effects. In comparison, glibenclamide produced a maximum reduction of 232 mg/dL, indicating that DCMOL and EAOL fractions exhibited comparable and in some doses even higher efficacy relative to the standard drug. Overall, the superior activity of DCMOL and EAOL suggests that bioactive constituents with intermediate polarity are primarily responsible for the observed antidiabetic effects as reported in Table 6.

**TABLE 6:** In vivo antidiabetic activity of *Oenothera laciniata* extract fractions compared to standard drug glibenclamide (GB).

S. no.	Groups	Dose mg/kg	Day 0th	Day 5th	Day 10th	Day15th	Decreased in blood glucose after 15 days mg/dL	Average change in body weight (g)
1	Normal Control	10 mL/kg	410.24 ± 1.12	332.56 ± 1.52	270.23 ± 0.91	270.45 ± 1.21	139	+3.12
2	Diabetic control	0.35 mL	481 ± 1.20	489 ± 2.20	498 ± 2.02	517 ± 1.34	−36	−13.7
3	Glibenclamide	10	392 ± 2.34***	333 ± 2.88***	289 ± 1.66***	160 ± 2.88***	232	+6.7
4	EAOL	75	431 ± 2.67***	407 ± 3.13***	387 ± 1.57***	296 ± 2.17***	135	+5.5
		150	410 ± 3.10**	340 ± 2.20***	320 ± 2.80***	271 ± 2.13***	139	
		300	425 ± 2.23**	395 ± 1.97***	341 ± 3.41***	218 ± 2.10***	207	
5	DCMOL	75	359 ± 2.20***	311 ± 1.90***	281 ± 2.45***	201 ± 2.30***	158	+4.1
		150	477 ± 1.02^ns^	301 ± 1.08***	261 ± 2.34***	275 ± 1.10***	202	
		300	460 ± 3.02***	296 ± 2.10***	260 ± 1.89***	235 ± 3.90***	225	
6	NHOL	75	389 ± 3.49*	357 ± 3.57^ns^	341 ± 3.41***	333 ± 3.21	56	+3.5
		150	468 ± 1.90^ns^	460 ± 4.50^ns^	440 ± 2.20*	405 ± 3.30	63	
		300	459 ± 2.29^ns^	404 ± 2.20*	359 ± 1.79	301 ± 1.20	158	
7	EOL	75	391 ± 3.97***	432 ± 2.30***	427 ± 3.31**	421 ± 2.21***	−30	−2.9
		150	441 ± 4.11***	417 ± 4.31***	410 ± 3.30**	401 ± 3.11***	40	
		300	439 ± 3.13***	418 ± 2.90***	398 ± 2.90***	381 ± 3.81***	58	
8	AQOL	75	353 ± 2.88***	338 ± 2.20***	305 ± 2.35***	288 ± 2.90***	65	+1.5
		150	448 ± 3.10*	427 ± 3.90**	393 ± 1.33***	367 ± 1.69***	81	
		300	428 ± 2.90**	417 ± 4.10***	386 ± 1.90***	338 ± 1.20***	90	

*Note:* Data are expressed as mean ± SEM (*n* = 3). Statistical analysis was performed using two‐way ANOVA followed by Bonferroni post‐test. Values are considered significant compared to the diabetic control group: *p < 0.05, **p < 0.01, ***p < 0.001; ns, not significant. Blood glucose levels are presented in mg/dL, and body weight changes are expressed in grams (g).

In addition to glycemic control, all treated groups exhibited notable effects on body weight, which is an important indicator of metabolic improvement in diabetic conditions. Glibenclamide‐treated rats showed the highest weight gain (6.7 g), whereas DCMOL, EAOL, NHOL, and AQOL groups demonstrated gains of 5.5, 4.1, 3.5, and 1.5 g, respectively. In contrast, EOL‐treated rats showed a slight weight loss (−2.9 g), which may reflect differences in phytochemical composition affecting energy metabolism and lipid utilization.

Alloxan‐induced diabetes is characterized by selective destruction of pancreatic β‐cells, resulting in insulin deficiency, oxidative stress, and sustained hyperglycemia (Talib et al. 2023). The significant reduction in blood glucose observed in this study indicates that the extracts may exert their effects through multiple complementary mechanisms, including (i) enhancement of residual pancreatic β‐cell function and insulin secretion, (ii) improvement of peripheral glucose uptake and utilization, and (iii) inhibition of carbohydrate‐hydrolyzing enzymes such as α‐amylase and α‐glucosidase, which collectively contribute to improved glycemic control. Similar findings have been reported in plant‐based antidiabetic studies where significant reductions in blood glucose were observed within 10–15 days of treatment in alloxan‐induced diabetic models, supporting the reproducibility and validity of the present results (Ovais et al. 2018). Furthermore, the marked activity of DCMOL and EAOL fractions can be attributed to the presence of phenolic and flavonoid‐rich phytoconstituents, which are well documented for their antidiabetic and antioxidant properties (Gul et al. 2025). These compounds have been reported to stimulate insulin secretion, enhance insulin sensitivity, and protect pancreatic β‐cells from oxidative damage. In addition, phenolic compounds are known to inhibit protein glycation and reduce oxidative stress, thereby preventing long‐term diabetic complications (Pannucci et al. 2023).

The overall antihyperglycemic effect observed in this study may therefore be explained by a synergistic dual mechanism involving enzyme inhibition and antioxidant‐mediated protection of pancreatic β‐cells (Ansari et al. 2023; Khan, Rahman, et al. 2025). By reducing oxidative stress and modulating carbohydrate metabolism, these extracts not only lower blood glucose levels but also potentially mitigate the progression of diabetes‐associated complications, including neuropathy, nephropathy, and retinopathy (Arunachalam et al. 2022; Shah et al. 2025). Collectively, these findings strongly suggest that *O. laciniata* , particularly its dichloromethane and ethyl acetate fractions, possesses significant therapeutic potential as a natural source of antidiabetic agents. However, future studies involving bioassay‐guided fractionation, compound isolation, and molecular docking validation are warranted to identify the exact active constituents and their precise molecular targets.

### 3.5 Change in Body Weight

**FIGURE 3:**
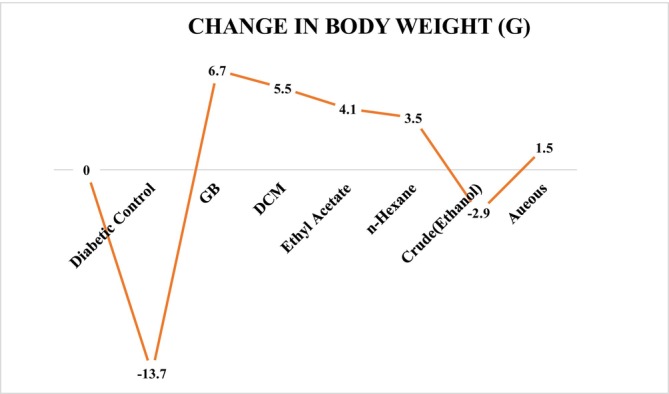
Effect of *Oenothera laciniata ext*racts on body weight in diabetic animals. Change in body weight (g) in diabetic control, GB (glibenclamide‐treated), DCM, ethyl acetate, n‐hexane, crude ethanolic, and aqueous extract‐treated groups. Positive values indicate weight gain, whereas negative values indicate weight loss compared to initial body weight.

The change in body weight across the treatment groups is shown in Figure 3. The untreated diabetic control group exhibited a marked reduction in body weight (−13.7 g), indicating severe metabolic impairment. In contrast, treatment with the standard drug GB (glibenclamide) resulted in a significant increase in body weight (+6.7 g), reflecting effective glycemic control. Among the crude and subsequent fractions treated groups, the DCM fraction (+5.5 g) and ethyl acetate fraction (+4.1 g) showed notable improvement, followed by the n‐hexane fraction (+3.5 g), all of which demonstrated partial recovery compared to the diabetic control. The aqueous fraction (+1.5 g) also improved body weight, although to a lesser extent than these fractions. In contrast, the crude ethanolic extract (−2.9 g) showed only limited protection against weight loss. Overall, while none of the extracts matched the effect of glibenclamide, the DCM and ethyl acetate fractions exhibited comparatively better efficacy in restoring body weight. The comparatively better efficacy of the DCM and ethyl acetate fractions in restoring body weight may be attributed to the nature of phytoconstituents enriched in these solvents (Abdullah et al. 2025). Semi‐polar fractions such as dichloromethane and ethyl acetate are known to concentrate bioactive compounds like flavonoids, phenolic acids, and certain terpenoids, which have been widely reported to possess antidiabetic and antioxidant properties. These compounds can improve glycemic control by enhancing peripheral glucose uptake, protecting pancreatic β‐cells from oxidative stress, and inhibiting carbohydrate‐digesting enzymes. Improved glycemic status reduces muscle wasting and prevents excessive breakdown of fats and proteins, thereby contributing to recovery of body weight (Aba and Asuzu 2018). In contrast, the crude ethanolic extract may contain a complex mixture of both active and less active constituents, potentially diluting the overall effect, whereas the aqueous fraction, although beneficial, may predominantly extract highly polar compounds with comparatively moderate activity.

### 3.6 Acute Toxicity Evaluation of Various Fractions of *O. laciniata* Extracts

Acute toxicity studies were conducted to determine the safe dose range of the tested fractions for in vivo analysis. No mortality or observable signs of toxicity were recorded in any treatment group during the 72‐h observation period, even at the highest tested dose of 1500 mg/kg body weight. These findings indicate that all tested fractions are relatively safe under the experimental conditions, with an LD_50_ value greater than 1500 mg/kg body weight.

### 3.7 Biochemical Assays of Various Fractions of *O. laciniata* Extracts

Biochemical estimation showed remarkable changes in liver and kidney function tests in alloxan‐induced diabetic rats (Group II), which were seen in the form of raised SGPT (51.22 ± 0.52 IU), SGOT (45.26 ± 1.20 IU), ALP (287.12 ± 0.10 IU), and serum creatinine (2.52 ± 0.02 mg/dL) reflecting hepatic and renal impairment (Table 7). Treatment with glibenclamide (Group III) restored these values significantly towards the normal value: SGPT (17.74 ± 0.71 IU), SGOT (19.11 ± 0.46 IU), ALP (176.16 ± 0.34 IU), and serum creatinine (0.534 ± 0.04 mg/dL). DCMOL extract (300 mg/kg) produced the best improvement, bringing down SGPT (21.02 ± 0.44 IU), SGOT (24.11 ± 0.07 IU), ALP (183.20 ± 0.02 IU), and creatinine (0.78 ± 0.08 mg/dL). EAOL at the same dosage markedly decreased SGPT (30.46 ± 0.43 IU), SGOT (27.52 ± 0.54 IU), and had intermediate improvement in ALP (137.64 ± 0.35 IU) and creatinine (1.61 ± 0.37 mg/dL). EOL extract decreased SGPT (37.54 ± 0.76 IU), SGOT (25.22 ± 0.20 IU), and ALP (167.34 ± 0.42 IU) but had minimal impact on creatinine. AQOL also improved at 300 mg/kg, reducing SGPT (36.34 ± 0.87 IU), SGOT (31.65 ± 0.22 IU), ALP (167.34 ± 0.42 IU), with insignificant effect on creatinine (1.54 ± 0.63 mg/dL). NHOL brought moderate hepatoprotection, significantly reducing SGPT and SGOT, but with little effect on ALP and not reducing creatinine levels (2.54 ± 0.43 mg/dL).

**TABLE 7:** Biochemical parameters following treatment with various fractions of *Oenothera laciniata* extracts.

Treatment	Dose	Biochemical changes after 15 days			
		SGPT IU	SGOT IU	ALP IU	Serum creatinine (mg/dL)
Group I Normal control	10 mL/kg	18.22 + 0.46***	20.33 + 0.43***	178.22 + 0.42***	0.541 + 0.06***
Group II Diabetic	150 mg/kg	51.22+ 0.52	45.26 + 1.20	287.12 + 0.10	2.52 + 0.02
Group III Glibenclamide	10 mg/kg	17.74 + 0.71***	19.11 + 0.46***	176.16 + 0.34***	0.534 + 0.04***
Group IV EOL	75 mg/kg	53.37 + 0.62***	35.68 + 0.76***	250.34 + 0.52^ns^	1.73 + 0.24***
	150 mg/kg	42.36 + 0.32***	30.22 + 0.20***	185.52 + 0.50***	2.66 + 0.10^ns^
	300 mg/kg	37.54 + 0.76***	25.22 + 0.20***	167.34 + 0.42***	2.54 + 0.43^ns^
Group V AQOL	75 mg/kg	55.26 + 0.82***	48.56 + 0.49***	276.44 + 0.22^ns^	1.83 + 0.04***
	150 mg/kg	47.56 + 0.67***	39.98 + 0.46***	234.21 + 0.62^ns^	2.34 + 0.04***
	300 mg/kg	36.34 + 0.87***	31.65 + 0.22***	167.34 + 0.42***	1.54 + 0.63^ns^
Group VI DCMOL	75 mg/kg	30.54 + 0.43***	27.52 + 0.54***	137.64 + 0.35***	1.61 + 0.37^ns^
	150 mg/kg	27.49 + 0.34***	29.87 + 0.91***	165.44 + 0.87***	0.84 + 0.67^ns^
	300 mg/kg	21.02 + 0.44***	24.11 + 0.07***	183.20 + 0.02***	0.78 + 0.08***
Group VII EAOL	75 mg/kg	51.55 + 0.57^ns^	24.68 + 0.74***	182.34 + 0.10***	0.75 + 0.02***
	150 mg/kg	46.64 + 0.76***	29.62 + 0.70***	169.54 + 0.64***	1.64 + 0.73^ns^
	300 mg/kg	30.46 + 0.43***	27.52 + 0.54***	137.64 + 0.35***	1.61 + 0.37^ns^
Group VIII NHOL	75 mg/kg	57.58 + 0.84***	32.89 + 0.54***	197.45 + 0.62***	1.76 + 0.95***
	150 mg/kg	48.98 + 0.67***	40.68 + 0.56***	245.26 + 0.62^ns^	1.52 + 0.24***
	300 mg/kg	37.54 + 0.76***	25.22 + 0.20***	167.34 + 0.42***	2.54 + 0.43^ns^

*Note:* Two‐way ANOVA followed by Bonferroni post‐test. Group II compared with Group III. After that group III compared with group (IV–VIII). Data are represented as changes in LFTs and serum creatinine (mean ± SEM of *n* = 3). *p < 0.05, **p < 0.01, ***p < 0.001. ns, not significant.

The effect of various plant extracts was evaluated on liver enzymes including SGPT (serum glutamate pyruvate transaminase), ALP (alkaline phosphatase), and SGOT (serum glutamate oxaloacetate transaminase). Biochemical assays revealed no significant alterations in serum levels of liver enzymes (SGPT, SGOT, and ALP) or creatinine in DCMOL, EAOL, and n‐hexane‐treated groups, indicating the extracts' safety at therapeutic doses (*p* > 0.05). However, the crude ethanolic extract slightly elevated serum creatinine levels, which necessitates caution in its use. Acute toxicity studies further confirmed the safety of the extracts, with no observed toxicity at doses up to 1500 mg/kg body weight.

### 3.8 Oral Glucose Tolerance Test for Various Fractions of *O. laciniata* Extracts

The OGTT evaluated antihyperglycemic activity of plant extracts in diabetic rats induced with streptozotocin by determining blood glucose at 0, 30, 60, and 120 min after glucose loading (Table 8). The diabetic control group (Group II) revealed impaired glucose tolerance with a peak at 120 min (311.6 ± 0.88 mg/dL), whereas the normal control group revealed a peak at 60 min (234.56 ± 0.68 mg/dL) and reverted towards baseline at 120 min (158.46 ± 2.52 mg/dL). Glibenclamide (Group III) inhibited glucose levels, peaking at 60 min (226.2 ± 1.46 mg/dL) and falling to 149.2 ± 1.34 mg/dL. The highest effect was observed with the DCMOL extract (Group VI) at 300 mg/kg with little increase at 60 min (221.2 ± 1.46 mg/dL) and at baseline at 120 min (149.9 ± 0.54 mg/dL). EOL (Group IV) exhibited dose‐dependent effects with 300 mg/kg lowering glucose to 176.8 ± 2.22 mg/dL at 120 min. EAOL (Group VII) at 300 mg/kg lowered glucose from 194.3 ± 2.02 mg/dL at 60 min to 159.9 ± 1.44 mg/dL at 120 min. AQOL (Group V) exhibited moderate activity, with 264.1 ± 1.88 mg/dL at 120 min at the maximum dose. NHOL (Group VIII) presented variable but significant glucose‐lowering, lowering to 165.3 ± 0.43 mg/dL at 120 min at 300 mg/kg. The OGTT results further corroborated the anti‐diabetic efficacy of the extracts. Overnight‐fasted rats were used for evaluation of oral glucose tolerance in both diabetic and treated animal models. The tested animals were administered glucose orally at the dose rate of 2 g/kg against the standard drug GB. Blood levels of the glucose were measured at 0, 30, 60, and 120 min after oral administration of glucose to evaluate the tolerance of rats to exogenous glucose. DCMOL‐treated rats exhibited a significant reduction in blood glucose levels (153.9 mg/dL) at 12 min post‐glucose load (*p* < 0.001), closely matching the effect of glibenclamide (149.2 mg/dL). EAOL and n‐hexane extracts also demonstrated promising glucose tolerance outcomes, whereas aqueous and crude ethanolic extracts showed comparatively weaker effects.

**TABLE 8:** Oral glucose tolerance test (OGTT) values for *Oenothera laciniata* fractions at varying dose levels.

Treatment	Dose (mg/kg)	OGTT (mg/dL)			
		0 min	30 min	60 min	120 min
Group‐I Normal control	10 mL/kg	162.4 ± 1.26***	192.38 ± 2.42***	234.56 ± 0.68**	158.46 ± 2.52***
Group‐II Diabetic	150	224.2 ± 1.20	243.2 ± 2.20	266.76 ± 1.10	311.6 ± 0.88
Group‐III Glibenclamide	10	159.6 ± 2.34***	186.3 ± 1.10***	226.2 ± 1.46**	149.2 ± 1.34***
Group‐IV EOL	75	198.76 ± 0.89***	233.4 ± 1.90***	256.3 ± 1.23**	196.1 ± 1.32***
	150	174.8 ± 1.80***	225.2 ± 0.90***	249.5 ± 1.16**	182.2 ± 1.12***
	300	171.3 ± 1.45***	199.4 ± 2.43***	229.3 ± 1.32**	176.8 ± 2.22***
Group‐V AQOL	75	201.34 ± 0.89***	243.7 ± 1.90***	266.3 ± 0.43**	199.2 ± 0.43***
	150	192.23 ± 0.45***	223.5 ± 0.44***	257.3 ± 1.23**	196.1 ± 1.32***
	300	188.2 ± 1.46***	193.7 ± 1.46***	221.6 ± 0.78^ns^	264.1 ± 1.88***
Group‐VI DCMOL	75	175.6 ± 0.67***	184.7 ± 0.58***	228.9 ± 0.72***	163.8 ± 0.55***
	150	165.3 ± 1.20***	167.8 ± 0.88***	237.3 ± 1.90***	153.9 ± 0.88***
	300	161.5 ± 0.34***	156.3 ± 1.10***	221.2 ± 1.46**	149.9 ± 0.54***
Group‐VII EAOL	75	197.56 ± 0.34***	231.6 ± 1.21***	261.4 ± 0.43**	186.2 ± 0.51***
	150	181.12 ± 0.45***	195.6 ± 0.44***	257.3 ± 1.23**	186.2 ± 0.42***
	300	171.3 ± 0.80***	188.3 ± 1.90***	194.3 ± 2.02***	159.9 ± 1.44***
Group‐VIII NHOL	75	211.4 ± 0.92***	198.2 ± 0.87***	254.6 ± 1.51***	199.1 ± 0.34***
	150	199.1 ± 0.72***	171.3 ± 1.91***	231.8 ± 0.32***	181.2 ± 0.11***
	300	182.9 ± 0.558***	199.5 ± 2.10***	226.1 ± 0.74**	165.3 ± 0.43***

*Note:* The data are represented as changes in blood glucose level (mean ± SEM of *n* = 3). Values were significantly different as compared to the diabetic control group (Tween 80). *p < 0.05, **p < 0.01, ***p < 0.001. ns, not significant.

### 3.9 Histopathology

The histopathological activities were investigated that Control rats showed normal islets of Langerhans with intact acini, whereas diabetic rats exhibited degeneration, fatty accumulation, and reduced islet size and number. Standard treatment restored islet and acinar architecture. Among extracts, DCMOL maintained near‐normal islet alignment with minimal fatty changes, whereas EAOL (300 mg/kg) promoted moderate regeneration. NHOL, EOL, and AQOL caused shrinkage with limited recovery. In the liver, controls displayed normal morphology, whereas diabetic rats showed fatty degeneration, vascular congestion, and inflammatory infiltrates. Standard treatment restored hepatocyte structure. DCMOL caused only mild fatty changes without necrosis, whereas EAOL showed partial recovery with residual fatty changes and inflammation. NHOL, EOL, and AQOL exhibited persistent damage. In the kidney, controls appeared normal, whereas diabetic rats exhibited inflammatory infiltrates and vascular congestion. Standard treatment restored renal morphology. DCMOL resulted in mild alterations without hyalinization, whereas EAOL caused infiltrates with hyalinization. NHOL, EOL, and AQOL showed persistent lesions. Comparable outcomes have been reported with other antidiabetic agents. Tatipamula et al. (2019) demonstrated that an indolizine derivative restored pancreatic islets, acini, and Sertoli cells in STZ‐induced diabetic rats. Similarly, Gawad et al. (2016) reported that bee venom therapy improved pancreatic islet architecture and hepatocyte integrity. These findings suggest that both natural phytoconstituents and synthetic derivatives exert antidiabetic effects partly through preservation and regeneration of vital tissues.

#### 3.9.1 Pancreas

After completing the compound treatment for 28 days, the group of animals was anesthetized by the ether and the abdomen of rats was dissected. Then the pancreas and testes were carefully dissected out and kept in saline and then stored in 10% formalin solution. The organs were sliced into 5 mm thick sections using a microtome, stained with hematoxylin and eosin, and used for histopathological examination.

**FIGURE 4:**
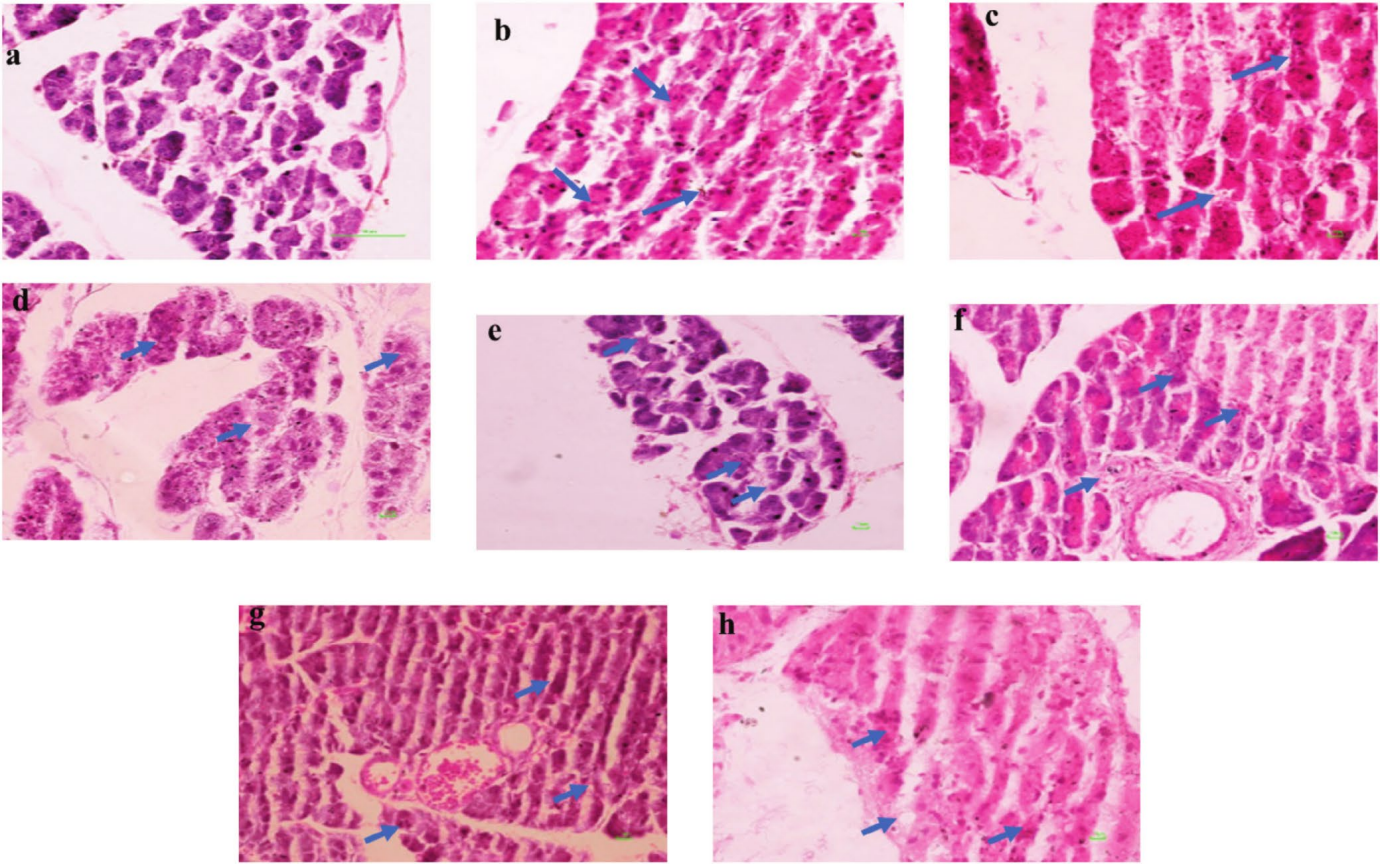
Histopathological investigations of pancreas: Normal control (a), diseased control (b), standard group (c), EAOL (d), DCM (e), NHOL (f), AQOL (g), and EOL (h). The histopathological investigations showed marked degeneration and accumulation of fat in the interlobular duct of the pancreas, damaged acini and islet cells, little fatty changes, shrinkage in islets of Langerhans, and congested blood vessels.

The pancrease of the all treated animals with highest doses were subjected to histopathological observations mentioned in Figure 4. The pancreatic sections were stained with hematoxylin and eosin and illustrated in figures, respectively. The histopathological examination of pancreas of control animals showed the well alignment of islets of Langerhans. There was no fatty change, necrosis, and congested blood vessels noticed in normal control group (Figure 4a) and the diabetic animal depicted the degeneration and accumulation of fat in interlobular duct of the pancreas and decline in size and number of acini and islets of Langerhans (Figure 4b). Besides, the standard treated animals revealed great recovery of damaged acini and islet cells (Figure 4c), whereas in extract EAOL with highest dose, that is, 300 administrated diabetic animals displayed the less regeneration of Langerhans islets and acini. Moreover, there was fatty changes from interlobular duct and also a clear reformation of intralobular duct were also observed (Figure 4d). Further, the number of islet cells and their diameter significantly augmented in these extracts and standard treated animals compared to diabetic group (Figure 4f). The histopathology of extract in DCMOL showed little fatty change and there was also well alignment of islet of Langerhans. There was no necrosis noticed (Figure 4g). Further extracts NHOL, EOL, and AQOL with highest doses there was prominent shrinkage in islets of Langerhans and, the decadence of fat from interlobular duct and also a clear reformation of intralobular duct were also observed.

#### 3.9.2 Liver

**FIGURE 5:**
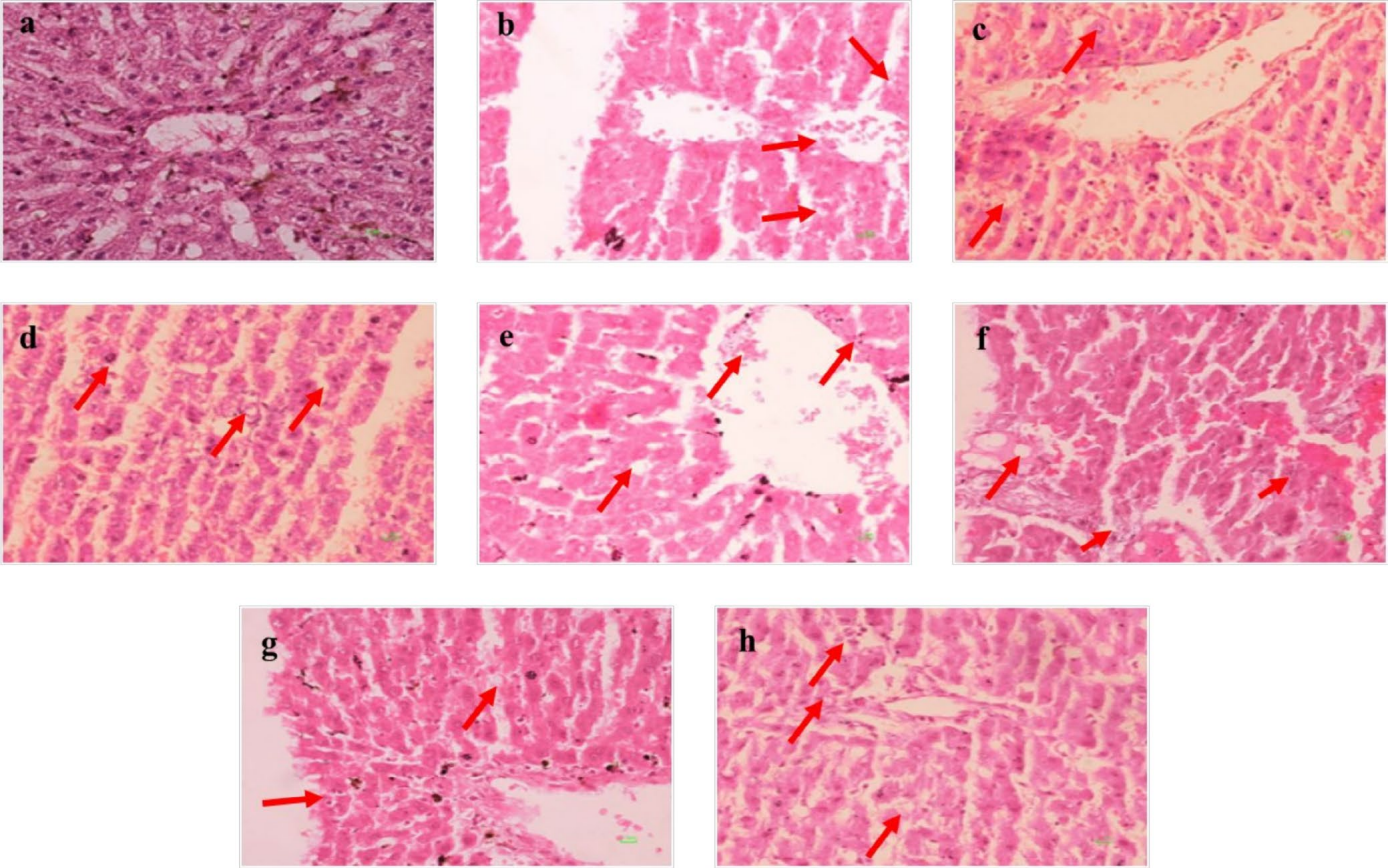
Histopathological investigations of liver: (a) Normal control, (b) diseased control, (c) standard group, (d) EAOL, (e) DCM, (f) NHOL, (g) AQOL, and (h) EOL. The histopathological investigations showed marked inflammatory infiltrates, cellular necrosis, little fatty changes, and congested blood vessels.

The liver of all treated animals was subjected to histopathological observations mentioned in Figure 5. The histopathological examination of the liver of control animals showed that there were no fatty changes, necrosis, congested blood vessels, or inflammatory infiltrate noticed in the normal control group (Figure 5a), and the diabetic animal depicted the accumulation of fat in the interlobular duct of the liver and inflammatory infiltrate. Further, blood vessels were congested but no necrosis was shown (Figure 5b). Besides, the standard treated animals revealed great recovery of damaged fatty changes with no necrosis and inflammatory infiltrate (Figure 5c), whereas in extract EAOL showed fatty changes and inflammatory infiltrate with the highest dose, that is, 300 mg/dL. Moreover, the decadence of fat from the interlobular duct and also a clear reformation of the intralobular duct were also observed (Figure 5d,e). The histopathology of extract DCMOL with the same highest dose showed little fatty change, and there was also no inflammatory infiltrate. The blood vessels were little congested. There was no necrosis noticed. Further, the remaining extracts NHOL, EOL, and AQOL showed prominent fatty changes, inflammatory infiltrate, and congested blood vessels.

#### 3.9.3 Kidney

**FIGURE 6:**
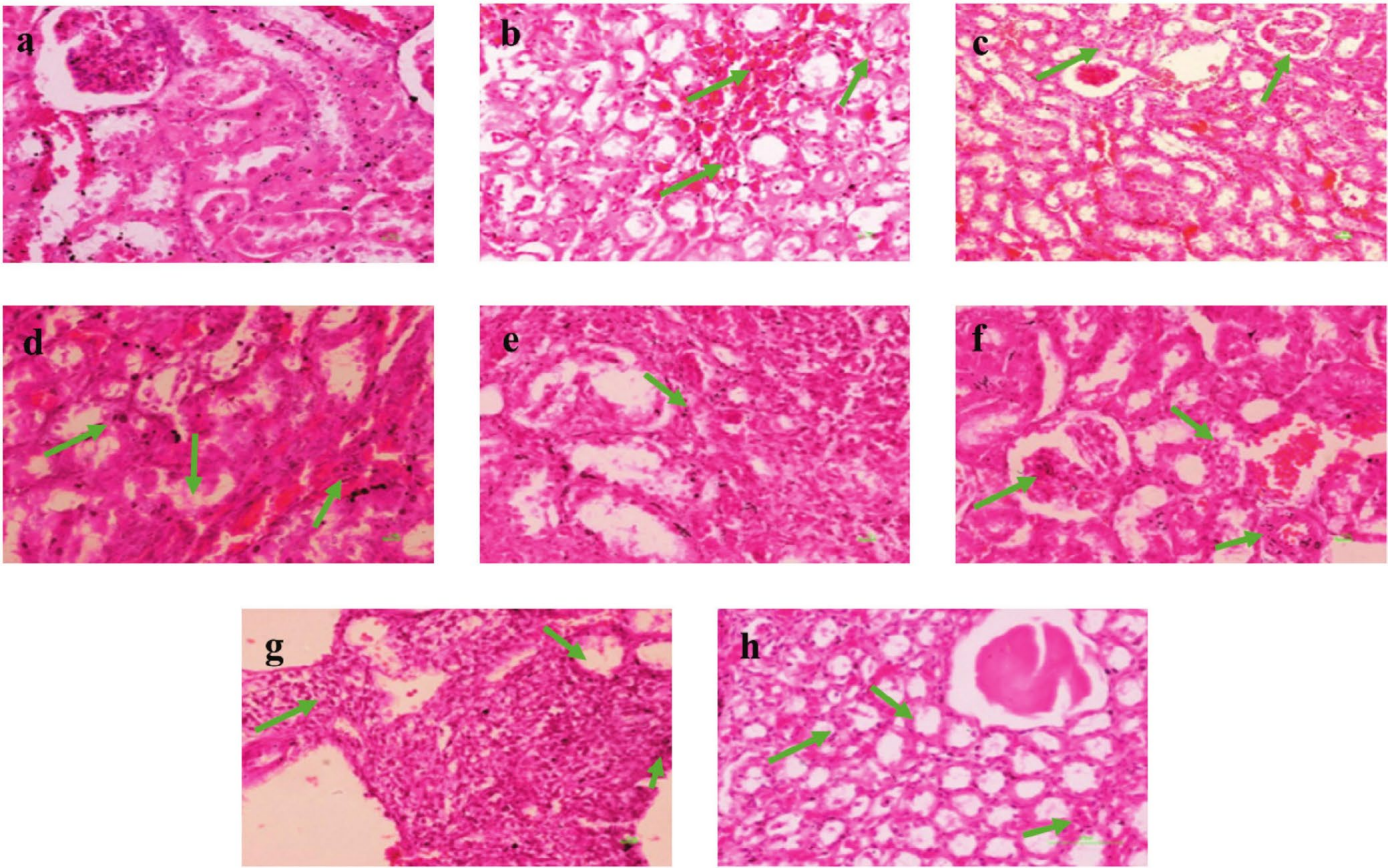
Histopathological investigations of kidney: (a) Normal control, (b) diseased control, (c) standard group, (d) EAOL, (e) DCM, (f) NHOL, (g) AQOL, and (h) EOL. The histopathological investigations showed marked inflammatory infiltrates, hyalinization, mild fatty changes, and moderate congested blood vessels.

The kidney of the all treated animals were subjected to histopathological screening mentioned in Figure 6. The histopathological examination of kidney of control animals showed that there was no inflammatory infiltrate, tubular degeneration, hyalinization and congested blood vessels noticed in normal control group (Figure 6a) and the diabetic animal depicted prominent inflammatory infiltrate. Furthermore, there were congested blood vessels as shown in Figure 6b. Besides, the standard treated animals revealed great recovery of damaged and inflammatory infiltrate and no hyalinization was noticed (Figure 6c), whereas extract EAOL showed inflammatory infiltrate, blood vessels, and hyalinization (Figure 6d). The histopathology of extract DCOM showed little inflammatory infiltrate. The blood vessels were little congested. There was no hyalinization noticed. Further extracts NHOL, EOL, and AQOL there were prominent inflammatory infiltrate and congested blood vessels were observed with highest dose.

### 3.10 Docking Studies

Docking simulations are the parameters which are adopted to analyze the binding affinities of a number of compounds against the specific proteins in a rapid and productive manner. These studies play a crucial role in understanding the behavior of natural as well as synthesized compounds, with macromolecular structures. In this study, GC–MS analysis fractions of *O. laciniata* were analyzed against glycosidase and alpha amylase to get the best outcome of pharmacological activities. The docking scores of different components have been tabulated in Tables 9 and 10. The docking parameters were performed in the presence of standard drug Acarbose so that results could be compared.

**TABLE 9:** Tabulating the binding affinities of GC–MS analysis fractions of *Oenothera laciniata* against glycosidase Pdb ID: 2ZEO.

Components	Binding affinity (kcal/mol)	RMSD lower bound	RMSD upper bound
2,2,4‐Trimethyl‐1,3‐pentanediol diisobutyrate	−6.3	7.757	11.215
9,12,15‐Octadecatrienoic acid, methyl ester (Z,Z,Z)‐	−5.9	4.412	7.099
9, 12‐Octadecadienoic acid (Z, Z)‐	−5.7	3.515	5.719
Bis(2‐ethylhexyl) phthalate	−6.6	2.532	6.901
Methyl 9‐cis,11‐trans‐octadecadienoate	−5.7	3.084	4.479
Octadecanoic acid	−5.2	3.639	6.437

**TABLE 10:** Tabulating the binding affinities of GC–MS analysis fractions of *Oenothera laciniata* against amylase Pdb ID: 3BC9.

Components	Binding affinity (kcal/mol)	RMSD lower bound	RMSD upper bound
2,2,4‐Trimethyl‐1,3‐pentanediol diisobutyrate	−6.4	3.545	7.589
9, 12, 15‐Octadecatrienoic acid, methyl ester (Z, Z, Z)‐	−5.3	19.209	21.597
9, 12‐Octadecadienoic acid (Z, Z)‐	−5.2	21.698	23.887
Bis(2‐ethylhexyl) phthalate	−5.5	20.079	22.773
Methyl 9‐cis,11‐trans‐octadecadienoate	−5.6	19.975	22.321
Octadecanoic acid	−4.9	17.542	21.147

**FIGURE 7:**
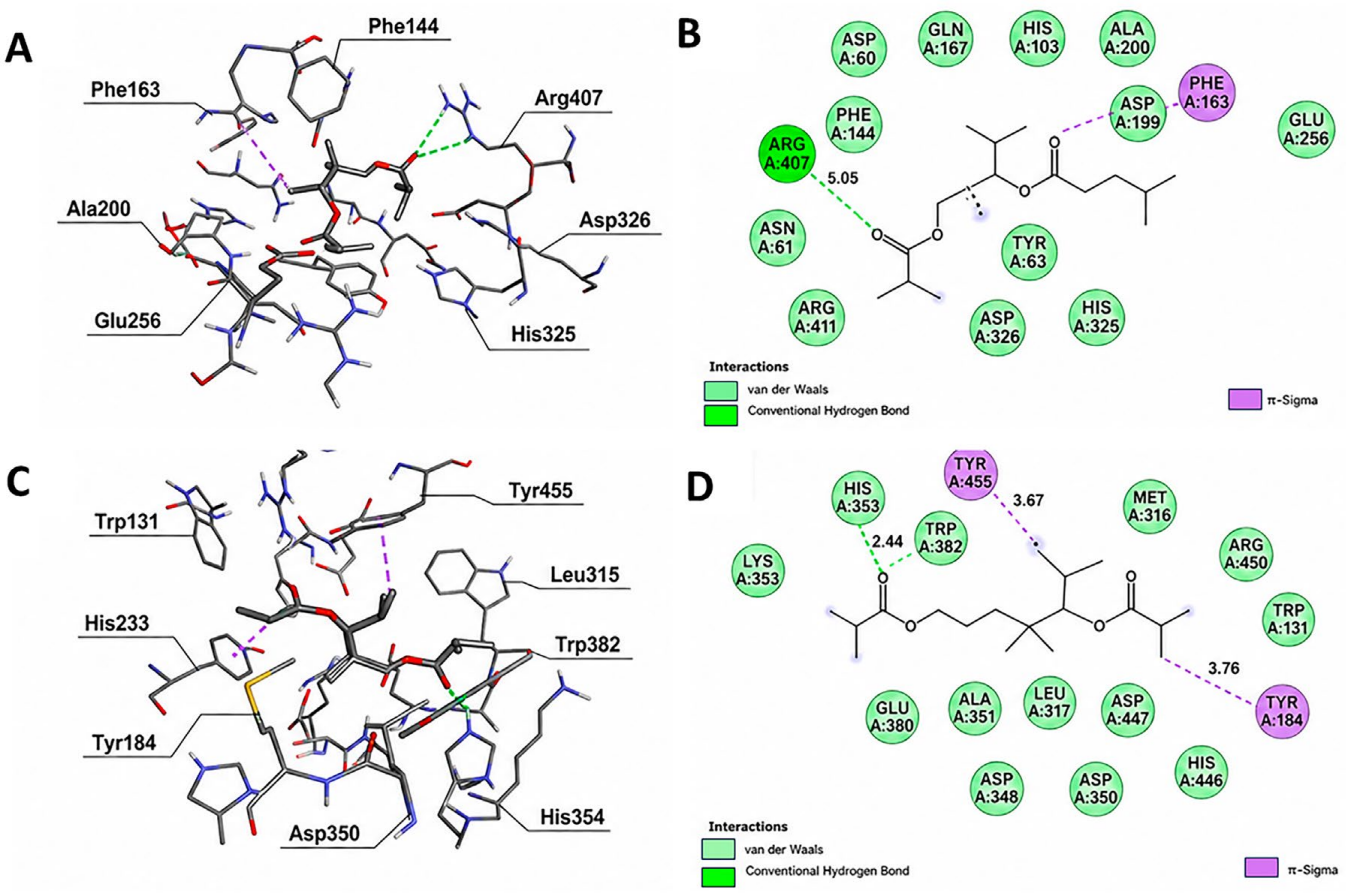
Displaying the docking results of 2, 2, 4‐trimethyl‐1, 3‐pentanediol diisobutyrate with targeted proteins (A) 3D image with glycosidase, (B) 2D image, (C) 3D image with alpha amylase, and (D) 2D image.

When visualizing the results of interactions of extracted component 2, 2, 4‐trimethyl‐1,3‐pentanediol diisobutyrate with glycosidase and alpha amylase, the output was satisfactory with binding energies of −6.3 and −6.4 kcal/mol. In case of glycosidase, it gave one conventional hydrogen bond with Arg407 with bond length of 5.05 Å and one pi‐sigma bond with Phe163 with bond length of 3.91 Å. Other amino acid AA residues were found to be His103, Ala200, Glu 256, His 325, Asp 326, and Arg 411. In the similar way with alpha amylase, it was found to give one conventional hydrogen bond with His354 with bond length of 2.02 Å, and two pi‐sigma bonds with Tyr455 and Tyr184 at the bond lengths of 3.67 and 3.76 Å, respectively. Other amino acid residues were Ala351, Leu315, Asp350, Arg348, His446, Glu380, Arg450, and Met316. The results have been displayed in Figure 7.

**FIGURE 8:**
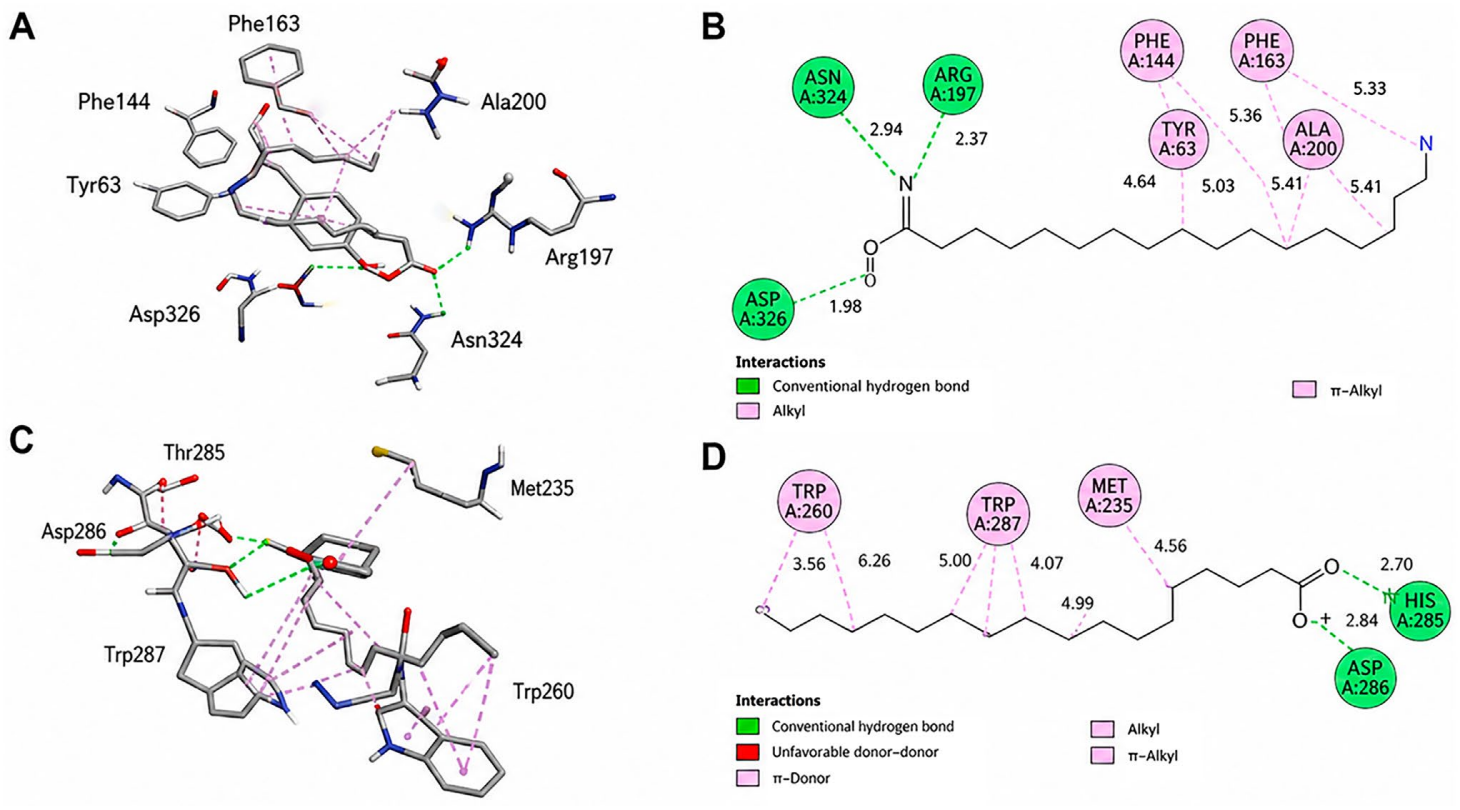
Displaying the docking results of 9, 12‐octadecadienoic acid (Z,Z)‐ with targeted proteins (A) 3D image with glycosidase, (B) 2D image, (C) 3D image with alpha amylase, and (D) 2D image.

When analyzing the results for other component as 9, 12‐octadecadienoic acid (Z,Z)‐, it showed satisfactory results with binding energies ranging from −5.7 to −5.2 kcal/mol for glycosidase and alpha amylase enzyme. In case of glycosidase, it was found to have three conventional hydrogen bonds with Asn324, Arg197, and Asp326, with bond lengths of 2.94, 2.37, and 1.98 Å respectively. Other active site residues were Phe144, Phe163, Tyr63, and Ala200. All the results have been displayed in Figure 8.

**FIGURE 9:**
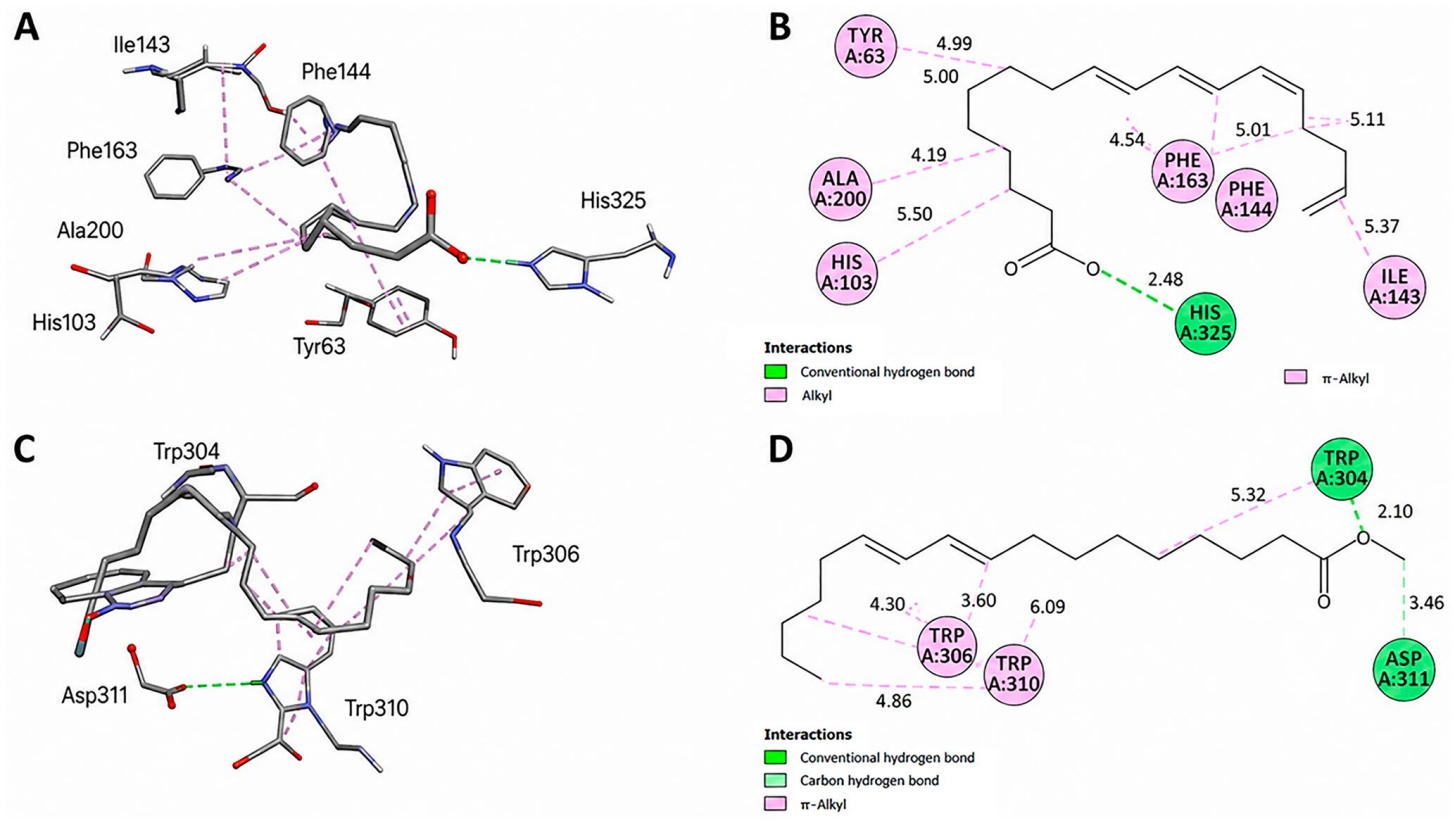
Displaying the docking results of methyl 9‐cis, 11‐trans‐octadecadienoate with targeted proteins (A) 3D image with glycosidase, (B) 2D image, (C) 3D image with alpha amylase, and (D) 2D image.

When examining the binding affinity of another important constituent of *O. laciniata* , that is, methyl 9‐cis, 11‐trans‐octadecadienoate, the results were satisfying with score in the range −5.7 to −5.6 kcal/mol for glycosidase and alpha amylase respectively. When discussing the interactions of glycosidase, the prominent binding was a conventional hydrogen bond with His325 at a bond length of 2.48 Å. Other amino acid residues in neighboring were Tyr63, Phe163, Phe144, Ile143, His103, and Ala200. In case of alpha amylase, it gave one conventional bond at the active site with Trp304 at a bond length of 2.10 Å, whereas other amino acids residues involved were Trp306, Trp310, and Asp311Figure 9.

## 4 Conclusion

The present study highlights the potent antidiabetic and antioxidant properties of various solvent extracts of *O. laciniata* , a plant known for its pharmacologically active phytoconstituents. Comprehensive GC–MS analysis of the extracts revealed the presence of key bioactive compounds, including Bis(2‐ethylhexyl) phthalate, octadecanoic acid, 9,12‐octadecadienoic acid, and γ‐sitosterol, which are largely responsible for the observed biological activities. Among the tested fractions, the dichloromethane extract (DCMOL) consistently showed the most promising results across all in vitro assays (DPPH, ABTS, H_2_O_2_ scavenging, α‐glucosidase, and α‐amylase inhibition), and also demonstrated the highest glucose‐lowering effects in vivo, followed closely by the ethyl acetate (EAOL) and n‐hexane (NHOL) extracts. Importantly, the extracts showed no significant toxicity at doses up to 1500 mg/kg, indicating a favorable safety profile. Additionally, the OGTT confirmed their effectiveness in managing postprandial blood glucose levels. Histopathological analysis of pancreas, liver, and kidney revealed severe degeneration, fatty changes, and inflammatory infiltrates in diabetic animals, whereas standard and some extract‐treated groups showed marked recovery and restoration of tissue architecture. Extracts exhibited variable protective effects, with EAOL and DCMOL demonstrating moderate regeneration, whereas NHOL, EOL, and AQOL showed limited recovery. These findings suggest that *O. laciniata* holds promising potential as a natural multi‐target therapeutic agent for the management of diabetes mellitus. However, the limitation of the current work is the primarily pharmacological evaluation based on preclinical in vitro and in vivo models, and the exact molecular mechanisms underlying the observed effects were not fully elucidated. In addition, isolation and characterization of individual active constituents responsible for the pharmacological activity were not performed. Future studies should focus on detailed mechanistic investigations, including molecular docking validation, enzyme kinetics, and pathway analysis, as well as isolation of pure bioactive compounds. Furthermore, long‐term toxicity studies and clinical investigations are necessary to validate the therapeutic potential and safety of *O. laciniata* in humans.

## Author Contributions

**Dur‐E‐Najaf Khan:** conceptualization, investigation. **Muhammad Saeed Jan:** conceptualization, investigation. **Syed Muhammad Mukarram Shah:** investigation, conceptualization, validation. **Muska Mahabat Khan:** conceptualization, investigation, writing – original draft. **Rehman Zafar:** writing – original draft, investigation. **Muhammad Ibrar:** conceptualization, investigation. **Yahya S. Al‐Awthan:** conceptualization, validation. **Omar S. Bahattab:** conceptualization, investigation. **Rakibur Rahman:** conceptualization, investigation. **Abdur Rauf:** conceptualization, writing – original draft.

## Funding

The authors have nothing to report.

## Consent

All the authors read and approved the final manuscript.

## Conflicts of Interest

The authors declare no conflicts of interest.

## Supporting information


**Figure S1:** Chromatogram of DCMOL sample.
**Figure S2:** Chromatogram of EAOL sample.
**Figure S3:** Chromatogram of NHOL sample.

## Data Availability

The data such as the source file associated with this finding are available from the corresponding author upon request.

## References

[fsn372095-bib-0001] Aba, P. E. , and I. U. Asuzu . 2018. “Mechanisms of Actions of Some Bioactive Anti‐Diabetic Principles From Phytochemicals of Medicinal Plants: A Review.” Indian Journal of Natural Products and Resources (IJNPR) 9, no. 2: 85–96.

[fsn372095-bib-0002] Abdullah, A. R. , M. A. Seliem , E. G. Khidr , A. M. Sobhy , R. A. El‐Shiekh , and M. S. A. E. Hafeez . 2025. “A Comprehensive Review on Diabetic Cardiomyopathy (DCM): Histological Spectrum, Diagnosis, Pathogenesis, and Management With Conventional Treatments and Natural Compounds.” Naunyn‐Schmiedeberg's Archives of Pharmacology 398, no. 8: 9929–9969. 40100371 10.1007/s00210-025-03980-9 PMC12350544

[fsn372095-bib-0003] Alhasaniah, A. H. , A. Sadiq , A. A. Al Awadh , Y. I. Asiri , M. Rezigue , and R. Zafar . 2025. “In‐Vitro, In‐Vivo and Computational Experiments for the Antidiabetic Potential of Bioactive Compound From *Notholirion thomsonianum* .” Natural Product Research: 1–14. 10.1080/14786419.2025.2509158 40434871

[fsn372095-bib-0004] Alkadi, H. 2020. “A Review on Free Radicals and Antioxidants.” Infectious Disorders Drug Targets 20, no. 1: 16–26. 29952268 10.2174/1871526518666180628124323

[fsn372095-bib-0005] Alshehri, O. M. , M. H. Mahnashi , A. Sadiq , R. Zafar , M. S. Jan , and F. Ullah . 2022. “Succinimide Derivatives as Antioxidant Anticholinesterases, Anti‐α‐Amylase, and Anti‐α‐Glucosidase: In Vitro and In Silico Approaches.” Evidence‐Based Complementary and Alternative Medicine 2022, no. 1: 6726438. 35942378 10.1155/2022/6726438 PMC9356783

[fsn372095-bib-0006] Alshehri, O. M. , M. Shabnam , S. A. Asiri , M. H. Mahnashi , A. Sadiq , and M. S. Jan . 2024. “Isolation, In Vitro, In Vivo Anti‐Inflammatory, Analgesic and Antioxidant Potential of *Habenaria plantegania* Lindl.” Inflammopharmacology 32, no. 2: 1353–1369. 38334860 10.1007/s10787-023-01425-4

[fsn372095-bib-0007] Al‐Snafi, A. E. 2020. “Oils and Fats Contents of Medicinal Plants, as Natural Ingredients for Many Therapeutic Purposes—A Review.” IOSR J. Pharm 10, no. 7: 1–41.

[fsn372095-bib-0008] Ansari, P. , J. F. Samia , J. T. Khan , M. R. Rafi , M. S. Rahman , and A. B. Rahman . 2023. “Protective Effects of Medicinal Plant‐Based Foods Against Diabetes: A Review on Pharmacology, Phytochemistry, and Molecular Mechanisms.” Nutrients 15, no. 14: 3266. 37513684 10.3390/nu15143266 PMC10383178

[fsn372095-bib-0009] Arunachalam, K. , P. S. Sreeja , and X. Yang . 2022. “The Antioxidant Properties of Mushroom Polysaccharides Can Potentially Mitigate Oxidative Stress, Beta‐Cell Dysfunction and Insulin Resistance.” Frontiers in Pharmacology 13: 874474. 35600869 10.3389/fphar.2022.874474 PMC9117613

[fsn372095-bib-0010] Asadi‐Kavan, Z. , R. A. Khavari‐Nejad , A. Iranbakhsh , and F. Najafi . 2020. “Cooperative Effects of Iron Oxide Nanoparticle (α‐Fe_2_O_3_) and Citrate on Germination and Oxidative System of Evening Primrose (*Oenthera biennis* L.).” Journal of Plant Interactions 15, no. 1: 166–179.

[fsn372095-bib-0011] Christopher, D. T. , I. E. Willie , I. O. Akpan , and E. T. Domingo . 2022. “Antioxidant Activity and Identification of Bioactive Compounds in *Telfairia occidentalis* Leaves Using GC–MS Analysis.” World Journal of Advanced Research and Reviews 16, no. 3: 775–788.

[fsn372095-bib-0012] Danesh, N. , S. Ansari , M.‐T. Golmakani , and M. Keramat . 2021. “Effect of Seaweed Extracts on Improving the Oxidation Kinetic of Black Cumin ( *Nigella sativa* ) Oil.” Journal of Applied Phycology 33, no. 1: 629–637.

[fsn372095-bib-0013] Eisa, N. , A. M. Masaad , A. A. Ahmed , A. Abubaker , A. Eisa , and M. Ahmed . 2026. “Chemical Composition GC/MS Analysis, Antioxidant and Antimicrobial Activity of Hibiscus, Baobab, and Buckhorn Seed Oil's.” Indonesian Journal of Chemical Analysis (IJCA) 9, no. 1: 68–79.

[fsn372095-bib-0014] Fan, J. , M. Ibrar , M. A. Khan , et al. 2025. “Phytochemical Screening and Antidiabetic Potential of *Paeonia emodi* in Alloxan‐Induced Diabetic Rats: Investigative Study Toward Possible Mechanism.” Food Science & Nutrition 13, no. 9: e70870. 40905017 10.1002/fsn3.70870 PMC12402602

[fsn372095-bib-0063] Gawad, S. A. , H. Fikry , M. M. Amin , A. R. Elmahdi , and D. Elaziz . 2016. “Effect of Apitherapy on the Pancreas and Liver of Streptozotacin Induced Diabetic Rats: A Biochemical and Histological Study.” European Journal of Pharmaceutical and Medical Research 3, no. 7: 555–565.

[fsn372095-bib-0015] Ghazi, D. , Z. Rasheed , and E. Yousif . 2018. “Review of Organotin Compounds: Chemistry and Applications.” Development 3, no. 4: 340–348.

[fsn372095-bib-0016] Gilbert, B. , L. F. Alves , and R. d. F. Favoreto . 2022. Monografias de Plantas Medicinais Brasileiras e Aclimatadas: Volume II. Editora Fiocruz.

[fsn372095-bib-0017] Gorlach, S. , W. Wagner , A. Podsedek , D. Sosnowska , J. Dastych , and M. Koziołkiewicz . 2011. “Polyphenols From Evening Primrose (*Oenothera paradoxa*) Defatted Seeds Induce Apoptosis in Human Colon Cancer Caco‐2 Cells.” Journal of Agricultural and Food Chemistry 59, no. 13: 6985–6997. 21627076 10.1021/jf200639e

[fsn372095-bib-0018] Goyal, S. , M. Sharma , and R. Sharma . 2022. “Bioactive Compound From *Lagerstroemia speciosa* : Activating Apoptotic Machinery in Pancreatic Cancer Cells.” 3 Biotech 12, no. 4: 96. 10.1007/s13205-022-03155-w PMC8933603 35371901

[fsn372095-bib-0019] Gul, S. , A. U. Rahman , F. Kausar , A. Iqbal , H. Gul , and M. S. Jan . 2025. “Phytochemical Profiling and Bioactivity of *Celtis caucasica* : Antioxidant, Antidiabetic, Anticholinesterase, and Anti‐Inflammatory Potential.” Scientific Reports 15: 20184. 10.1038/s41598-025-06171-y.40542038

[fsn372095-bib-0020] Hsu, C.‐C. , H.‐C. Kuo , and K.‐E. Huang . 2017. “The Effects of Phytosterols Extracted From *Diascorea alata* on the Antioxidant Activity, Plasma Lipids, and Hematological Profiles in Taiwanese Menopausal Women.” Nutrients 9, no. 12: 1320. 29206136 10.3390/nu9121320 PMC5748770

[fsn372095-bib-0021] Hussain, A. 2020. “A Preliminary Up‐to‐Date Review on Pakistani Medicinal Plants With Potential Antioxidant Activity.” RADS Journal of Biological Research & Applied Sciences 11, no. 1: 61–88.

[fsn372095-bib-0022] Hussain, F. , A. Tahir , M. S. Jan , N. Fatima , A. Sadiq , and U. Rashid . 2024. “Exploitation of the Multitarget Role of New Ferulic and Gallic Acid Derivatives in Oxidative Stress‐Related Alzheimer's Disease Therapies: Design, Synthesis and Bioevaluation.” RSC Advances 14, no. 15: 10304–10321. 38549798 10.1039/d4ra00766b PMC10976477

[fsn372095-bib-0023] Imtiaz, F. , M. Islam , H. Saeed , A. Ahmed , and H. A. Rathore . 2023. “Assessment of the Antidiabetic Potential of Extract and Novel Phytoniosomes Formulation of *Tradescantia pallida* Leaves in the Alloxan‐Induced Diabetic Mouse Model.” FASEB Journal 37, no. 4: e22818. 36856606 10.1096/fj.202201395RR PMC11977607

[fsn372095-bib-0024] Jacob, B. , and R. Narendhirakannan . 2019. “Role of Medicinal Plants in the Management of Diabetes Mellitus: A Review.” 3 Biotech 9, no. 1: 4. 10.1007/s13205-018-1528-0 PMC6291410 30555770

[fsn372095-bib-0025] Jan, A. , M. Saeed , R. A. Mothana , T. Muhammad , N. Rahman , and A. R. Alanzi . 2023. “Association of CYP2C9* 2 Allele With Sulphonylurea‐Induced Hypoglycaemia in Type 2 Diabetes Mellitus Patients: A Pharmacogenetic Study in Pakistani Pashtun Population.” Biomedicine 11, no. 8: 2282. 10.3390/biomedicines11082282 PMC10452755 37626778

[fsn372095-bib-0026] Kaur, N. , L. Vij , T. Kaur , and M. S. Bhullar . 2025. “Alterations in the Primary Metabolite Profiles of Field Dodder ( *Cuscuta campestris* ) and Its Associated Hosts Cutleaf Evening Primrose ( *Oenothera laciniata* ) and Swine Cress ( *Coronopus didymus* ) in the Fields of North‐West India.” Indian Society of Weed Science 57, no. 1: 9489.

[fsn372095-bib-0027] Khan, A. , A. Pervaiz , B. Ansari , R. Ullah , S. M. M. Shah , and H. Khan . 2022. “Phytochemical Profiling, Anti‐Inflammatory, Anti‐Oxidant and In‐Silico Approach of *Cornus macrophylla* Bioss (Bark).” Molecules 27, no. 13: 4081. 35807324 10.3390/molecules27134081 PMC9268425

[fsn372095-bib-0028] Khan, A. , A. Pervaiz , M. S. Jan , B. Ansari , I. Ahmad , and S. M. M. Shah . 2025. “Assessment of Preclinical Antioxidative and Anti‐Inflammatory Activities of *Cornus macrophylla* Wall. Bark.” Food Science & Nutrition 13, no. 7: e70620. 40672543 10.1002/fsn3.70620 PMC12264324

[fsn372095-bib-0029] Khan, A. A. , F. U. Rahman , M. Shabnam , F. Hussain , M. S. Jan , and A. U. Rahman . 2025. “Phytochemical Profiling and Dual In Vitro In Vivo Antidiabetic Assessment of Ethyl Acetate Fraction and Essential Oil From *Allium ascalonicum* .” Biomedical Chromatography 39, no. 10: e70193. 40820276 10.1002/bmc.70193

[fsn372095-bib-0030] Koba, K. , and T. Yanagita . 2014. “Health Benefits of Conjugated Linoleic Acid (CLA).” Obesity Research & Clinical Practice 8, no. 6: e525–e532. 25434907 10.1016/j.orcp.2013.10.001

[fsn372095-bib-0031] Kwak, C. S. , M.‐J. Kim , S. G. Kim , S. Park , I. G. Kim , and H. S. Kang . 2019. “Antioxidant and Antiobesity Activities of Oral Treatment With Ethanol Extract From Sprout of Evening Primrose ( *Oenothera laciniata* ) in High Fat Diet‐Induced Obese Mice.” Journal of Nutrition and Health 52, no. 6: 529–539.

[fsn372095-bib-0032] Mahmood, F. , R. Ali , M. S. Jan , K. A. Chishti , S. Ahmad , and A. Zeb . 2019. “Chemical Characterization and Analgesic Potential of *Notholirion thomsonianum* Extract.” Latin American Journal of Pharmacy 38, no. 4: 807–812.

[fsn372095-bib-0033] Mahnashi, M. H. , W. Alam , M. A. Huneif , A. Abdulwahab , M. J. Alzahrani , and K. S. Alshaibari . 2023. “Exploration of Succinimide Derivative as a Multi‐Target, Anti‐Diabetic Agent: In Vitro and In Vivo Approaches.” Molecules 28, no. 4: 1589. 36838577 10.3390/molecules28041589 PMC9964140

[fsn372095-bib-0034] Mahnashi, M. H. , Y. S. Alqahtani , B. A. Alyami , A. O. Alqarni , M. Ahmed Alshrahili , and M. A. Abou‐Salim . 2022. “GC–MS Analysis and Various In Vitro and In Vivo Pharmacological Potential of *Habenaria plantaginea* Lindl.” Evidence‐Based Complementary and Alternative Medicine 2022, no. 1: 7921408. 35399645 10.1155/2022/7921408 PMC8989558

[fsn372095-bib-0035] Mahnashi, M. H. , B. A. Alyami , Y. S. Alqahtani , A. O. Alqarni , M. S. Jan , and M. Ayaz . 2022. “Molecular Docking Supported Observed Changes in Anticholinesterase, Antioxidant and α‐Glucosidase Inhibitions Upon the Bromination of Benzene Sulfonamide.” Journal of the Chemical Society of Pakistan 44, no. 1: 69.

[fsn372095-bib-0036] Mahnashi, M. H. , U. Rashid , H. H. Almasoudi , M. H. Nahari , I. Ahmad , and A. S. Binshaya . 2024. “Modification of 4‐(4‐Chlorothiophen‐2‐Yl)thiazol‐2‐Amine Derivatives for the Treatment of Analgesia and Inflammation: Synthesis and In Vitro, In Vivo, and In Silico Studies.” Frontiers in Pharmacology 15: 1366695. 10.3389/fphar.2024.1366695. 38487174 PMC10937574

[fsn372095-bib-0037] Masood, N. , Q. Jamil , M. I. Aslam , M. I. Masood , J. H. Shirazi , and Q. A. Jamil . 2023. “Antioxidant, Carbonic Anhydrase Inhibition and Diuretic Activity of *Leptadenia pyrotechnica* Forssk. Decne.” Heliyon 9, no. 12: e22485. 38076186 10.1016/j.heliyon.2023.e22485 PMC10709400

[fsn372095-bib-0038] Mirzaei, A. , B. Nasr Esfahani , M. Ghanadian , and S. Moghim . 2022. “ *Alhagi maurorum* Extract Modulates Quorum Sensing Genes and Biofilm Formation in *Proteus mirabilis* .” Scientific Reports 12, no. 1: 13992. 35978046 10.1038/s41598-022-18362-x PMC9385855

[fsn372095-bib-0039] Munir, R. , N. Semmar , M. Farman , and N. S. Ahmad . 2017. “An Updated Review on Pharmacological Activities and Phytochemical Constituents of Evening Primrose (Genus *Oenothera*).” Asian Pacific Journal of Tropical Biomedicine 7, no. 11: 1046–1054.

[fsn372095-bib-0040] Nema, R. , and S. Khare . 2020. “In Vitro Cytotoxic Activity Toward Anticancer and Antimicrobial of *Azadirachta indica* , *Aegle marmelos* , *Ocimum sanctum* and *Withania somnifera* Extracts.” Bioprocess Engineering 4, no. 2: 40–46.

[fsn372095-bib-0041] Ovais, M. , M. Ayaz , A. T. Khalil , et al. 2018. “HPLC‐DAD Finger Printing, Antioxidant, Cholinesterase, and α‐Glucosidase Inhibitory Potentials of a Novel Plant *Olax nana* .” BMC Complementary and Alternative Medicine 18, no. 1: 1. 29295712 10.1186/s12906-017-2057-9 PMC5751879

[fsn372095-bib-0042] Pannucci, E. , L. Spagnuolo , L. De Gara , L. Santi , and L. Dugo . 2023. “Phenolic Compounds as Preventive and Therapeutic Agents in Diabetes‐Related Oxidative Stress, Inflammation, Advanced Glycation End‐Products Production and Insulin Sensitivity.” Discovery Medicine 35, no. 178: 715–732. 37811611 10.24976/Discov.Med.202335178.68

[fsn372095-bib-0043] Pervaiz, A. , M. S. Jan , S. M. Hassan Shah , A. Khan , R. Zafar , and B. Ansari . 2022. “Comparative In‐Vitro Anti‐Inflammatory, Anticholinesterase and Antidiabetic Evaluation: Computational and Kinetic Assessment of Succinimides Cyano‐Acetate Derivatives.” Journal of Biomolecular Structure and Dynamics 41, no. 18: 1–14. 10.1080/07391102.2022.2069862 35507043

[fsn372095-bib-0044] Ratan, Y. , A. Rajput , A. Pareek , A. Pareek , R. Kaur , and S. Sonia . 2024. “Recent Advances in Biomolecular Patho‐Mechanistic Pathways Behind the Development and Progression of Diabetic Neuropathy.” Biomedicine 12, no. 7: 1390. 10.3390/biomedicines12071390 PMC11273858 39061964

[fsn372095-bib-0045] Rauf, A. , N. Almasoud , M. Ibrahim , T. S. Alomar , A. A. Khalil , and T. Khursheed . 2024. “Anti‐Diabetic, Anti‐Cholinesterase, and Anti‐Inflammatory Potential of Plant Derived Extracts and Column Semi‐Purified Fractions of *Ficus benghalensis* .” Frontiers in Bioscience (Landmark Edition) 29, no. 5: 183. 38812295 10.31083/j.fbl2905183

[fsn372095-bib-0046] Rauf, A. , M. Ibrahim , T. S. Alomar , N. AlMasoud , A. A. Khalil , and M. Khan . 2024. “Hypoglycemic, Anti‐Inflammatory, and Neuroprotective Potentials of Crude Methanolic Extract From *Acacia nilotica* L.—Results of an In Vitro Study.” Food Science & Nutrition 12, no. 5: 3483–3491. 38726429 10.1002/fsn3.4017 PMC11077208

[fsn372095-bib-0047] Sadiq, A. , M. H. Mahnashi , U. Rashid , M. S. Jan , M. A. Alshahrani , and M. A. Huneif . 2022. “3‐(((1 S, 3 S)‐3‐((R)‐Hydroxy (4‐(Trifluoromethyl) Phenyl) Methyl)‐4‐Oxocyclohexyl) Methyl) Pentane‐2, 4‐Dione: Design and Synthesis of New Stereopure Multi‐Target Antidiabetic Agent.” Molecules 27, no. 10: 3265. 35630740 10.3390/molecules27103265 PMC9146474

[fsn372095-bib-0048] Saeedi, P. , I. Petersohn , P. Salpea , B. Malanda , S. Karuranga , and N. Unwin . 2019. “Global and Regional Diabetes Prevalence Estimates for 2019 and Projections for 2030 and 2045: Results From the International Diabetes Federation Diabetes Atlas.” Diabetes Research and Clinical Practice 157: 107843. 31518657 10.1016/j.diabres.2019.107843

[fsn372095-bib-0049] Sazdova, I. , M. Keremidarska‐Markova , D. Dimitrova , V. Mitrokhin , A. Kamkin , and N. Hadzi‐Petrushev . 2023. “Anticarcinogenic Potency of EF24: An Overview of Its Pharmacokinetics, Efficacy, Mechanism of Action, and Nanoformulation for Drug Delivery.” Cancers 15, no. 22: 5478. 38001739 10.3390/cancers15225478 PMC10670065

[fsn372095-bib-0050] Shah, L. , A. Iqbal , M. Imran , M. S. Jan , E. R. Elsharkawy , and Z. Shah . 2025. “Phytochemical Profiling and Bioactive Potential of *Rhizoclonium hookeri* , Antioxidant, Antidiabetic, and Neuroprotective Effects.” Chemistry & Biodiversity 22, no. 11: e00947. 40789071 10.1002/cbdv.202500947

[fsn372095-bib-0051] Shah, M. , M. S. Jan , A. Sadiq , S. Khan , and U. Rashid . 2023. “SAR and Lead Optimization of (Z)‐5‐(4‐Hydroxy‐3‐Methoxybenzylidene)‐3‐(2‐Morpholinoacetyl) Thiazolidine‐2, 4‐Dione as a Potential Multi‐Target Antidiabetic Agent.” European Journal of Medicinal Chemistry 258: 115591. 37393789 10.1016/j.ejmech.2023.115591

[fsn372095-bib-0052] Sidiq, S. S. , Q. Jabeen , Q. Jamil , M. S. Jan , I. Iqbal , and F. Saqib . 2025. “Lemongrass Alleviates Primary Dysmenorrhea Symptoms by Reducing Oxidative Stress and Inflammation and Relaxing the Uterine Muscles.” Antioxidants 14, no. 7: 838. 40722942 10.3390/antiox14070838 PMC12291959

[fsn372095-bib-0053] Sunil, J. , Y. K. Janapati , and S. S. Junapudi . 2024. “The Classical Biomarkers to Predict Diabetes Mellitus.” Asian Journal of Medical Research and Health Sciences (A‐JMRHS) 2, no. 1: 5–8.

[fsn372095-bib-0054] Talib, A. , S. A. Shah , M. S. Jan , M. Z. Ahsan , A. Munir , and I. A. Bukhari . 2023. “Exploration of Ketone Derivatives of Succinimide for Their Antidiabetic Potential: In Vitro and In Vivo Approaches.” Green Processing and Synthesis 12, no. 1: 20230103.

[fsn372095-bib-0062] Tatipamula, V. B. , M. K. Kolli , S. B. Lagu , K. R. Paidi , P. R. Reddy , and R. P. Yejella . 2019. “Novel Indolizine Derivatives Lowers Blood Glucose Levels in Streptozotocin‐Induced Diabetic Rats: A Histopathological Approach.” Pharmacological Reports 71, no. 2: 233–242. 30807980 10.1016/j.pharep.2018.11.004

[fsn372095-bib-0055] Tiwana, G. , I. E. Cock , and M. J. Cheesman . 2025. “ *Phyllanthus emblica* : Phytochemistry, Antimicrobial Potential With Antibiotic Enhancement, and Toxicity Insights.” Microorganisms 13, no. 3: 611. 40142504 10.3390/microorganisms13030611 PMC11945131

[fsn372095-bib-0056] Tortosa‐Caparrós, E. , D. Navas‐Carrillo , F. Marín , and E. Orenes‐Piñero . 2017. “Anti‐Inflammatory Effects of Omega 3 and Omega 6 Polyunsaturated Fatty Acids in Cardiovascular Disease and Metabolic Syndrome.” Critical Reviews in Food Science and Nutrition 57, no. 16: 3421–3429. 26745681 10.1080/10408398.2015.1126549

[fsn372095-bib-0057] Ullah, S. , S. A. Halim , M. Waqas , F. Mansoor , S. K. Avula , and F. A. Khan . 2025. “Biochemical and Computational Inhibition of α‐Glucosidase by Novel Metronidazole‐Linked 1 H‐1, 2, 3‐Triazole and Carboxylate Moieties: Kinetics and Dynamic Investigations.” Journal of Biomolecular Structure and Dynamics 43, no. 13: 6749–6769. 38433423 10.1080/07391102.2024.2322622

[fsn372095-bib-0058] Waheed, B. , S. M. Mukarram Shah , F. Hussain , M. I. Khan , A. Zeb , and M. S. Jan . 2022. “Synthesis, Antioxidant, and Antidiabetic Activities of Ketone Derivatives of Succinimide.” Evidence‐Based Complementary and Alternative Medicine 2022, no. 1: 1445604. 35388310 10.1155/2022/1445604 PMC8979682

[fsn372095-bib-0059] Widyawati, P. S. , T. D. W. Budianta , F. A. Kusuma , and E. L. Wijaya . 2014. “Difference of Solvent Polarity to Phytochemical Content and Antioxidant Activity of *Pluchea indicia* Less Leaves Extracts.” International Journal of Pharmacognosy and Phytochemical Research 6, no. 4: 850–855.

[fsn372095-bib-0060] Yoon, W.‐J. , Y. M. Ham , B.‐S. Yoo , J.‐Y. Moon , J. Koh , and C.‐G. Hyun . 2009. “ *Oenothera laciniata* Inhibits Lipopolysaccharide Induced Production of Nitric Oxide, Prostaglandin E2, and Proinflammatory Cytokines in RAW264.7 Macrophages.” Journal of Bioscience and Bioengineering 107, no. 4: 429–438. 19332304 10.1016/j.jbiosc.2008.11.018

[fsn372095-bib-0061] Yousefi, F. , S. Mahjoub , M. Pouramir , and F. Khadir . 2013. “Hypoglycemic Activity of *Pyrus biossieriana* Buhse Leaf Extract and Arbutin: Inhibitory Effects on Alpha Amylase and Alpha Glucosidase.” Caspian Journal of Internal Medicine 4, no. 4: 763–767. 24294470 PMC3841776

