# Supplementary material for: Exploring the Therapeutic Potential of Oenothera laciniata: Antioxidant, Anti‐Hyperglycemic and Histopathological Activities Using In Vitro and In Vivo Approaches
Source: Food Sci Nutr. 2026 Jul 13;14(7):e72095. doi: 10.1002/fsn3.72095 (PMC13359343; doi:10.1002/fsn3.72095)
Supplement: Supplementary file 1 — Figure S1: Chromatogram of DCMOL sample. Figure S2: Chromatogram of EAOL sample. Figure S3: Chromatogram of NHOL sample. [file FSN3-14-e72095-s001.docx]

**Exploring the Therapeutic Potential of *Oenothera laciniata*: Antioxidant, Anti-Hyperglycemic and Histopathological Activities Using *in-vitro* and *in-vivo* approaches**

Dur-E-Najaf Khan^1,2^, Syed Muhammad Mukarram Shah^1^, Muska Mahabat Khan^1^, Rehman Zafar^3^, Muhammad Saeed Jan^2^, Muhammad Ibrar^1^, Abdur Rauf^4^, Yahya S. Al-Awthan^5,6^, Omar S. Bahattab^5^, Rakibur Rahman^7^

^1^Department of Pharmacy, University of Swabi, KP, Pakistan

^2^Department of Pharmacy, Bacha Khan University, Charsadda, KP, Pakistan

^3^Akson College of Pharmacy, Mirpur University of Science and Technology, Mirpur, AJ& Kashmir

^4^Department of Chemistry, University of Swabi, Swabi, Anbar 23561, Khyber Pakhtunkhwa, Pakistan

^5^Department of Biology, Faculty of Science, University of Tabuk, Tabuk 71491, Saudi Arabia

^6^Biodiversity Genomics Unit, Faculty of Science, University of Tabuk, Tabuk, 71491, Saudi Arabia

^7^Department of Pharmacy, Faculty of Science and Engineering, International Islamic University Chittagong, Chattogram-4318, Bangladesh

**
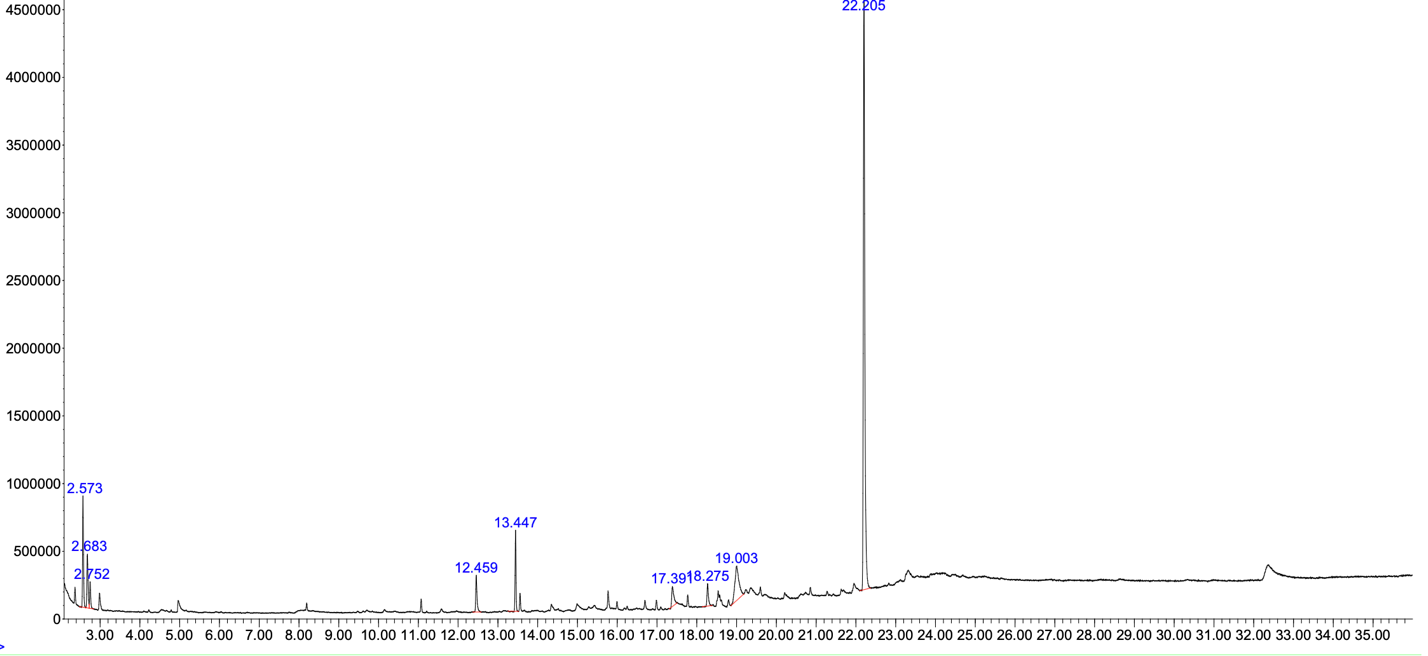
**

**Figure S1.** Chromatogram of DCMOL sample.

**
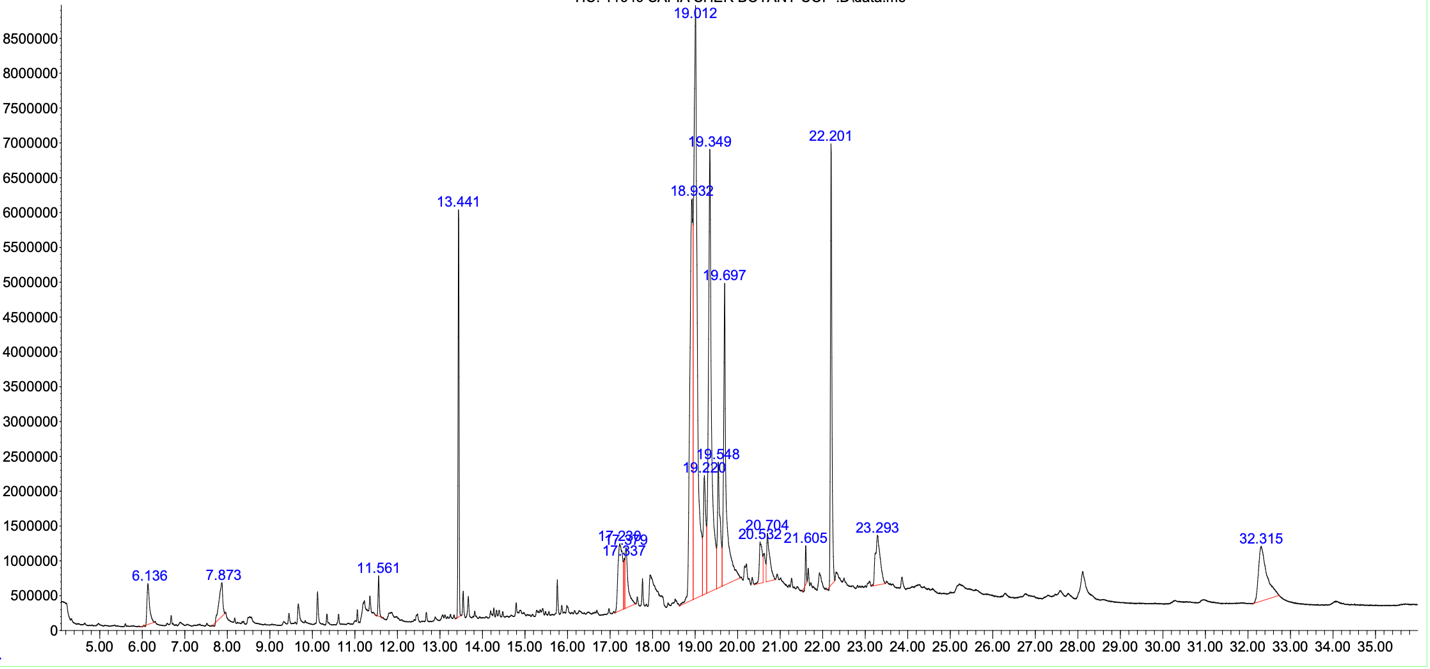
**

**Figure S2.** Chromatogram of EAOL sample.


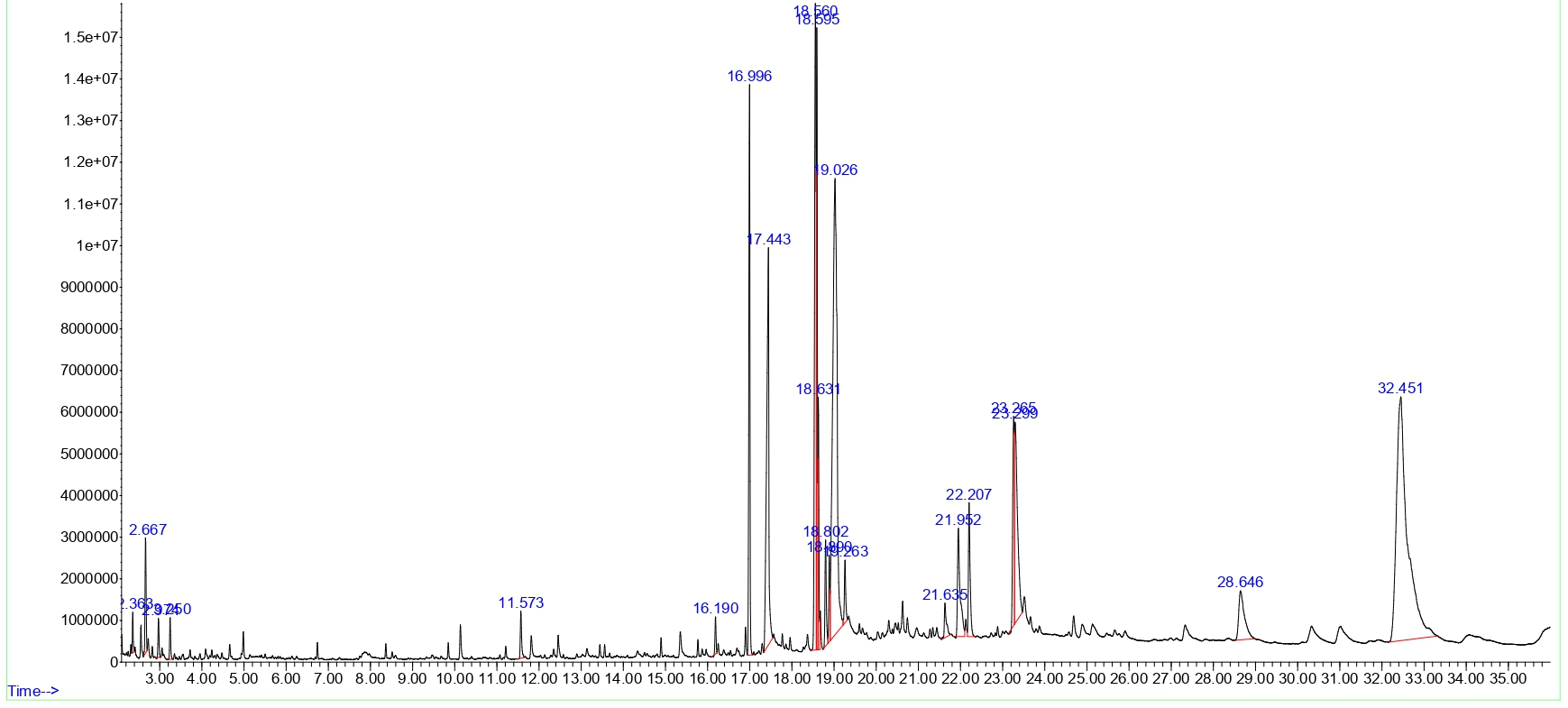


**Figure S3.** Chromatogram of NHOL sample.
